# Urinary Polycyclic Aromatic Hydrocarbon Metabolites Are Associated with Biomarkers of Chronic Endocrine Stress, Oxidative Stress, and Inflammation in Adolescents: FLEHS-4 (2016–2020)

**DOI:** 10.3390/toxics9100245

**Published:** 2021-10-01

**Authors:** Veerle J. Verheyen, Sylvie Remy, Eva Govarts, Ann Colles, Laura Rodriguez Martin, Gudrun Koppen, Stefan Voorspoels, Liesbeth Bruckers, Esmée M. Bijnens, Stijn Vos, Bert Morrens, Dries Coertjens, Annelies De Decker, Carmen Franken, Elly Den Hond, Vera Nelen, Adrian Covaci, Ilse Loots, Stefaan De Henauw, Nicolas Van Larebeke, Caroline Teughels, Tim S. Nawrot, Greet Schoeters

**Affiliations:** 1 VITO Health, Flemish Institute for Technological Research (VITO), Boeretang 200, 2400 Mol, Belgium; sylvie.remy@vito.be (S.R.); eva.govarts@vito.be (E.G.); ann.colles@vito.be (A.C.); laura.rodriguezmartin@vito.be (L.R.M.); gudrun.koppen@vito.be (G.K.); greet.schoeters@vito.be (G.S.); 2 Department of Biomedical Sciences, University of Antwerp, Universiteitsplein 1, 2610 Wilrijk, Belgium; 3 VITO GOAL, Flemish Institute for Technological Research (VITO), Boeretang 200, 2400 Mol, Belgium; stefan.voorspoels@vito.be; 4 I-BioStat, Data Science Institute, Hasselt University, Martelarenlaan 42, 3500 Hasselt, Belgium; Liesbeth.bruckers@uhasselt.be; 5 Centre for Environmental Sciences, Hasselt University, Agoralaan Building D, 3590 Diepenbeek, Belgium; esmee.bijnens@uhasselt.be (E.M.B.); stijn.vos@uhasselt.be (S.V.); tim.nawrot@uhasselt.be (T.S.N.); 6 Department of Sociology, Faculty of Social Sciences, University of Antwerp, Sint-Jacobstraat 2, 2000 Antwerp, Belgium; bert.morrens@uantwerpen.be (B.M.); dries.coertjens@uantwerpen.be (D.C.); ilse.loots@uantwerpen.be (I.L.); 7 Provincial Institute of Hygiene, Kronenburgstraat 45, 2000 Antwerp, Belgium; Annelies.DEDECKER@provincieantwerpen.be (A.D.D.); Carmen.Franken@provincieantwerpen.be (C.F.); elly.denhond@provincieantwerpen.be (E.D.H.); vera.NELEN@provincieantwerpen.be (V.N.); 8 Toxicological Centre, University of Antwerp, Universiteitsplein 1, 2610 Wilrijk, Belgium; adrian.covaci@uantwerpen.be; 9 Department of Public Health and Primary Care, Faculty of Medicine and Health Sciences, Ghent University, De Pintelaan 185, 9000 Ghent, Belgium; stefaan.dehenauw@ugent.be; 10 Analytical, Environmental and Geo-Chemistry, Vrije Universiteit Brussel, 1050 Brussels, Belgium; nicolas.vanlarebeke@ugent.be; 11 Department of Radiotherapy and Experimental Cancerology, Ghent University, B-9000 Ghent, Belgium; 12 Flemish Planning Bureau for the Environment and Spatial Development, Koning Albert II laan 20, bus 8, 1000 Brussels, Belgium; caroline.teughels@vlaanderen.be

**Keywords:** adolescents, human biomonitoring, Flemish Environment and Health Study (FLEHS), polycyclic aromatic hydrocarbons, hair cortisol concentration, neutrophil–lymphocyte ratio, 8-oxo-7,8-dihydro-2’-deoxyguanosine

## Abstract

Polycyclic aromatic hydrocarbons (PAHs) are environmental pollutants of public health concern. Multiple biological mechanisms have been hypothesized to contribute to PAHs-associated adverse health effects. Little is known about the impact of PAHs on endocrine stress and inflammation in adolescence. We examined 393 Flemish adolescents (14–15 years) cross-sectionally, measured urinary concentrations of hydroxylated naphthalene, fluorene, phenanthrene and pyrene metabolites, and calculated the sum of all measured metabolites. We determined hair cortisol concentration (HCC) as endocrine stress biomarker, leucocyte counts and neutrophil–lymphocyte ratio (NLR) in peripheral blood as inflammatory biomarkers, and urinary 8-oxo-7,8-dihydro-2’-deoxyguanosine (8-oxodG) concentration as oxidative stress biomarker. Exposure–response associations were analyzed by multiple regression, adjusted for a priori selected covariates. A doubling of 1-hydroxypyrene concentration was associated with a factor of 1.13 (95% CI: 1.03, 1.24) increase in HCC and a factor of 1.07 (95% CI: 1.02, 1.13) increase in 8-oxodG. Doublings of 2- and 3-hydroxyphenanthrene concentrations were associated with a factor of 1.08 (95% CI: 1.02, 1.14) and 1.06 (95% CI: 1.00, 1.12) increase in 8-oxodG, respectively. Doubling of 2-hydroxyphenanthrene and of the sum of 2- and 3-hydroxyfluorene was associated with, respectively, a factor of 1.08 (95% CI: 1.02, 1.14) and 1.06 (95% CI: 1.01, 1.13) increase in NLR. Our results indicate the glucocorticoid pathway as a potential target for PAH exposure in adolescents and suggest oxidative stress, endocrine stress, and inflammation in adolescence as underlying mechanisms and early markers for PAH-related adverse health effects.

## 1. Introduction

Polycyclic aromatic hydrocarbons (PAHs) are a large class of widespread environmental pollutants generated by incomplete combustion of organic materials, such as coal, oil, wood, and gas [1]. The general population is exposed to PAHs through inhalation of polluted ambient air from a variety of sources such as motor vehicles, residential heating, industry and environmental tobacco smoke, through ingestion of contaminated food, grilled and smoked food, and to a lesser extent, through dermal contact [2,3]. In the air, PAHs with a low molecular weight are mainly present in the vapor phase, while those with a high molecular weight mainly bind to particles [4]. After entering the body, PAHs are readily metabolized and eliminated in urine [5]. Urinary concentrations of hydroxylated PAHs, metabolic products of PAHs, reflect the integrated exposure through inhalation, ingestion, and dermal uptake in the hours to days prior to sampling [6].

Environmental exposure to PAHs has primarily been linked to cancer, in particular skin and lung, bladder, and gastrointestinal cancers [7,8]. Furthermore, a growing body of scientific evidence suggests that environmental exposure to PAHs may play a role in the development of chronic health conditions, such as adverse respiratory, cardiovascular, and cognitive outcomes [9,10,11,12,13]. PAHs have also been shown to be potent immune suppressants [5]. Due to their toxicity and ubiquity in the environment, PAHs are a global public health concern [14].

The biological mechanisms that contribute to PAHs-associated chronic health effects are still under investigation but are likely to be multifactorial. Toxicity of PAHs may in part be caused by reactive PAH intermediates that are formed during metabolism and that generate oxidative stress, which in turn can result in systemic inflammation and chronic disease pathogenesis [2,13]. In epidemiological studies, oxidative stress is a well-studied mechanism of downstream PAH toxicity in different age groups [15,16,17,18]. Reactive PAH intermediates may also form covalent adducts with nucleic acid and lead to genotoxic effects [8]. Furthermore, PAHs are suspected endocrine disruptors [1,19]. In this context, PAHs have been suggested to disrupt the endocrine glucocorticoid (GC) homeostasis, possibly resulting in long-term increases in circulating levels of GCs, a hallmark of chronic endocrine stress [1,20]. The hypothalamic–pituitary–adrenal (HPA) axis, with the GC cortisol as its main effector, is a crucial biological stress response system in humans [21]. Chronic endocrine stress has been linked to increased risk of cardiovascular and respiratory diseases and reduced cognitive function [22]. Cortisol also has well-known immunomodulatory effects [23]. Oxidative stress, inflammation, and endocrine stress are interrelated biological mechanisms [24,25] and may each individually or jointly underlie PAHs-associated adverse chronic health outcomes.

Importantly, the health effects of environmental exposures also depend on the time in the life span when exposure occurs. Adolescence is a developmental period with increased vulnerability to environmental exposures [26]. The ongoing maturation of the endocrine, immune, and central nervous systems makes adverse effects of environmental exposures on these systems during adolescence particularly harmful [22,23]. Significant associations between PAH exposure, oxidative stress, and inflammation have previously been reported in adolescents [6,17]. To the best of our knowledge, chronic endocrine stress has not been investigated in relation to PAHs exposure in any age group. Chronic endocrine stress during adolescence has implications for physical and mental health that reach into adulthood [27].

The aim of this study, conducted in the framework of the fourth Flemish Environment and Health Study (2016–2020), was to investigate associations of urinary OH-PAHs with early pathophysiological mechanisms that may underlie PAHs-associated health effects in a general population of Flemish adolescents, in particular chronic endocrine stress, inflammation, and oxidative stress.

We hypothesized that internal exposure to PAHs is associated with an increase in chronic endocrine stress in adolescents, together with simultaneous increases in oxidative stress and inflammation. 

Traditional assessment of HPA axis function through measurement of cortisol levels in saliva, blood, or urine reflects cortisol concentrations over minutes to 24 h prior to sampling. Longer-term cortisol concentration, as an indicator of chronic endocrine stress, is difficult to evaluate using these matrices due to circadian variations in cortisol secretion and the need for multiple sampling [28]. The hair cortisol concentration (HCC) of a single scalp-near hair sample has been shown to retrospectively reflect circulating cortisol levels and chronic endocrine stress over a period of several months prior to sampling [29,30]. The validity of HCC as a biomarker of long-term circulating cortisol concentrations was demonstrated in direct and indirect validation studies [31]. Strong positive associations between HCC and mean salivary cortisol levels, obtained through repeated sampling, were first established in animals and confirmed in humans [32,33]. In experimental animal studies, repeated stimulation of cortisol secretion by administration of HPA axis hormones (adrenocorticotropic hormone, corticotropin-releasing hormone) was associated with an increased accumulation of cortisol in hair [34]. In humans, research has also associated HCC with conditions that are known to be related to altered adrenocortical function, such as Cushing’s syndrome, Addison’s disease, and cardiovascular diseases [35]. A systematic review [30] identified sex, age, anthropometry, and household socio-economic status (SES) as determinants of HCC in adolescents. With regard to HCC as a biomarker of perceived stress, results have been mixed [31]. On balance, available evidence suggests that HCC is a suitable biomarker for chronic endocrine stress reflected in long-term circulating cortisol concentration; evidence for HCC as a biomarker of perceived stress is less substantial.

Total and differential leucocyte counts in peripheral blood are widely-used hematological biomarkers of inflammation [36] and have been used as an immunologic end point to detect immunotoxic effects of environmental contaminants in epidemiologic studies in adolescents [37,38]. Leucocytes signal clinically relevant hematologic changes that may result in clinically identifiable immune disorders, with absolute numbers providing biologically more reliable information than differential leukocyte percentages [39]. The combined neutrophil–lymphocyte ratio (NLR) is a clinically used inflammatory biomarker that correlates well with increased severity of infections, respiratory and cardiovascular diseases [40,41] that may provide information on relevant immunologic changes. The urinary concentration of 8-oxo-7,8-dihydro-2’-deoxyguanosine (8-oxodG), an oxidized nucleoside of DNA, is an established biomarker of oxidative stress following chemical exposure and was included as an oxidative stress biomarker in relation to PAH exposure in adolescents in previous FLEHS campaigns [17].

We simultaneously evaluated associations of urinary concentrations of PAHs metabolites in Flemish adolescents (14–15 years) with (1) the cortisol concentration in a 3 cm scalp-near hair segment, reflecting circulating cortisol levels in a 3-month period prior to sampling; (2) total and differential leucocyte count (neutrophils, lymphocytes, and monocytes) and NLR in peripheral blood; (3) urinary 8-oxodG concentration. Given the widespread environmental exposure to PAHs and the vulnerability of adolescents to PAH-associated adverse health effects, a better insight into early pathophysiological mechanisms that may be related to PAH exposure in adolescents is of global public health importance.

## 2. Materials and Methods

### 2.1. Study Population

This study was embedded in the fourth Flemish Environment and Health study (FLEHS-4), a human biomonitoring study including 428 adolescents from the general population of Flanders. The FLEHS-4 study protocol was approved in June 2017 by the Antwerp University Hospital Ethics committee (Belgian registration number B300201732753). A stratified clustered multi-stage sampling strategy was applied to enroll equal numbers of participants across both sexes and to represent all educational levels and all Flemish provinces proportionally to the general Flemish population. The inclusion criteria of the FLEHS-4 study were informed consent signed by the adolescent and a parent, lived in Flanders for at least 5 years, and adolescent and parents mastered enough Dutch to fill out extensive questionnaires. Exclusion criteria were data of more than one questionnaire missing, blood and urine sample missing, being held back in school for more than one year, or attending a boarding school. Adolescents that attend boarding school spend a large part of their time away from home and are therefore less representative of environmental exposures in the residential surroundings. One participant was excluded from FLEHS-4 because of pregnancy. FLEHS-4 participants were excluded from the study if no data were available of urinary PAH-metabolites, urinary specific gravity, leucocyte count, or HCC. One participant was excluded from the study because of following a growth hormone therapy and a second because of a clinically elevated leucocyte count (>20,000 cells/µL). The final study population consisted of 393 adolescents. The study was conducted according to the criteria set by the declaration of Helsinki [42].

### 2.2. Data Collection

Between September 2017 and June 2018, participants were examined in their schools by trained nurses. Weight and height were measured using calibrated equipment. From this information, the body mass index (BMI) was calculated. Boys and girls were classified as underweight, normal weight, overweight, or obese according to the sex- and age-specific 2004 Belgian growth curves [43]. Beforehand, participants and their parents filled out extensive questionnaires with information on lifestyle and health. Parents reported their perceived income adequacy, a subjective measure of household socio-economic status (SES) ranging from difficult to easy and very easy to make ends meet. Research has shown subjective income measures to be associated with health, above and beyond the health benefits associated with objective income measures [44]. On the day of examination, adolescents filled in an additional questionnaire, including information on smoking (yes/no), residential exposure to environmental tobacco smoke (ETS) (yes/no), recent health complaints (any health complaint within fourteen days prior to sampling), and recent medication use (i.e., use of systemic glucocorticoids). During the examination, a spot urine sample, a blood sample, and a scalp-near hair sample of at least three centimeters were obtained.

Data on the local ambient temperature at the time of sampling were provided by the Belgian Royal Meteorological Institute (KMI) that measures temperature in 15 weather stations across Flanders. We used data from the weather station closest to the adolescents’ homes. Based on the participants’ geocoded home addresses, we assessed the Area Deprivation Index (ADI) at the sub-municipality level as an indicator of neighborhood SES, as previously described [45]. 

### 2.3. Analysis of Urinary PAH Metabolites and Urinary Specific Gravity

Spot urine samples were collected during field work in clean polyethylene (PE) containers and were processed immediately. To determine the specific gravity of the urine samples, 2 mL was aliquoted in a polypropylene tube, kept at 4 °C, and analyzed within 48 h after collection by refractometry at the accredited medical laboratory A.M.L. (Algemeen Medisch Laboratorium, Antwerp, Belgium). For analysis of urinary levels of hydroxylated metabolites (OH-PAHs), 2.5 mL of urine was aliquoted in polypropylene corning tubes, subsequently frozen and stored at −20 °C until analysis. We measured urinary levels of hydroxylated metabolites of four PAHs, from here on referred to as OH-PAHs. The analyzed OH-PAHs, with their limits of quantification (LOQ), were the pyrene metabolite 1-hydroxypyrene (1-OHPy, 0.015 µg/L), the naphthalene metabolite 2-hydroxynaphthalene (2-OHNa, 0.150 µg/L), the sum of fluorene metabolites 2- and 3-hydroxyfluorene (2,3-OHFl, 0.030 µg/L) and phenanthrene metabolites 2-hydroxyphenanthrene (2-OHPH, 0.015 µg/L), 3-hydroxyphenanthrene (3-OHPH, 0.014 µg/L), 4-hydroxyphenanthrene (4-OHPH, 0.014 µg/L), and the sum of 1- and 9-hydroxyphenanthrene (1,9-OHPH, 0.031 µg/L).

The analysis was performed at the Flemish Institute for Technological Research (VITO GOAL, Mol, Belgium), using liquid chromatography coupled to mass spectrometry (UPLC–MS/MS). First, 500 μL of urine was hydrolyzed overnight at 37 °C with β-glucuronidase/arylsulfatase in the presence of a sodium acetate buffer (pH = 5) to obtain unconjugated OH-PAHs. A constant amount of internal standards for each analyte (i.e., 13C-1-OHPy_,_ 13C-2-OHNa, 13C-2-OHFl, 13C-3-OHPH, 13C-4-OHPH, d8-9-OHPH) was added to the mixture to correct for the loss of analyte during sample preparation. Internal standards were provided by Campro Scientific GmbH, Veenendaal, the Netherlands, except for 13C-1-OHPy, which was provided by Sigma-Aldrich, Overijse, Belgium. After incubation, 240 µL acetonitrile was added to the mixture to keep the OH-PAHs in solution. An aliquot was transferred to an LC-vial and analyzed using ultra-high pressure liquid chromatography (UPLC) coupled to a tandem mass spectrometer (Xevo TQ-S, Waters, MA, USA) equipped with an Acquity BEH C18 column (2.1 × 100 mm, 1.7 µm; Waters, MA, USA). Mass spectrometry was performed in negative ESI MRM mode, and two mass transitions were registered per analyte. All analytical lab operations were performed according to ISO 17025 from sample registration to reporting. The analytical method used was fully validated. Shewart charts were used to control quality over the total duration of the project. Warning limits were used as a trigger for reanalysis of the entire batch of the day. Practical internal quality control consisted of different measures. Analysis of blanks ensured absence of false positives. Random replicate sample analysis was used to control precision. In general, the relative standard deviation (RSD, *n* = 2) ranged between 0 and 15%, where the higher values were recorded for samples with concentrations close to the LOQ. Repeated random analysis of spiked samples and of a control sample with standard reference material SRM 3672 [46], containing organic contaminants including OH-PAHs in smokers’ urine, were used to control trueness. An average recovery of 91–99% (RSD 2–10%, *n* = 23) was hereby recorded. External quality control was assured through successful participation in the Human Biomonitoring for Europe External Quality Assurance Scheme (HBM4EU ICI/EQUAS) for PAH metabolites [47].

### 2.4. Analysis of 8-oxo-7,8-dihydro-2’-deoxyguanosine (8-oxodG) in Urine

For the determination of 8-oxodG, 1 mL urine was aliquoted, subsequently frozen, and stored at −80 °C until analysis at VITO Health. After thawing, samples were centrifuged at 2000× *g* for 15 min. Fifty μL of the supernatant was used to determine 8-oxodG, using a competitive enzyme-linked immunosorbent assay (ELISA) kit (Japan Institute for the Control of Aging, Shizuoka, Japan), according to manufacturer’s instructions. The determination range was 0.5–200 ng/mL. The anti-8-oxodG mouse monoclonal antibody (clone N45.1), with an established specificity, was used as a primary antibody [48]. The values from each urine sample were calculated based on calibration sigmoid plots of absorbance (450 nm) of an 8-oxodG standard at various concentrations. External quality control was assured through participation in an international ring test [49].

### 2.5. Hair Sample Collection and Hair Cortisol Measurement

During fieldwork, a strand of hair of at least three centimeters was cut close to the scalp from the posterior vertex of the adolescents’ head. Hair samples were stored in paper envelopes at room temperature until analysis, within 18 months after the collection of the first samples. When protected from ultraviolet light, cortisol concentrations in hair samples remain stable at room temperature for several years [50]. Cortisol levels were determined at the Institute of Public Health, Department of Environmental Medicine of the University of Southern Denmark (SDU) from the 3 cm scalp-near hair segment. Human scalp hair grows at a speed of approximately 1 cm per month, HCC of 3 cm hair strands retrospectively reflect cortisol levels for the period of three months [32]. Samples were washed with methanol to eliminate exogenous contamination, dried at room temperature, and analyzed using High-Performance Liquid Chromatography (HPLC) combined with tandem mass spectrometry (HPLC–MS/MS), as previously described [45]. HPLC was performed using an Accella 1250 pump (Thermo Scientific, San Jose, CA, USA) and a PAL autosampler (CTC Analytics, Zwingen, Switzerland). The analytical column was a Kinetex C18 column, 100 × 4.6 mm (2.6 μm), equipped with a 2 × 4 mm C18 Security Guard column (Phenomenex, Torrance, CA, USA). The triple quadrupole mass spectrometer utilized was a TSQ Vantage (Thermo Scientific, San Jose, CA, USA). Quality control samples were included in the analytical sequence. The limit of quantification (LOQ) for cortisol and cortisone was 0.3 pg/mg hair. The intra-day repeatability was 8.7%, and the inter-day precision was 9.5%.

### 2.6. Total and Differential Leucocyte Count in Peripheral Blood

A blood sample of 6 mL was collected in an EDTA tube and gently mixed; 1 mL was then aliquoted in a Sarstedt tube. Samples were stored at 4 °C until analysis within 48 h after collection at A.M.L. Leucocyte count and leucocyte subtype distribution (percentage) were assessed using a Sysmex XE-2100 instrument for hematology analysis (Sysmex Corporation, Kobe, Japan), a widely used automated hematology system that combines flow cytometry with fluorescence detection, using a diode laser bench [51]. The neutrophil–lymphocyte ratio was calculated by dividing the neutrophil count by the lymphocyte count.

### 2.7. Statistical Analysis

Statistical analysis was performed using SPSS Statistics (version 27; IBM, Armonk, NY, USA) and R version 3.5.0 (R Foundation for Statistical Computing, Vienna, Austria). OH-PAHs concentrations, HCC, and 8-oxodG concentrations below the LOQ were imputed with a random value (between 0 and the LOQ), drawn from the estimation of the lognormal distribution of all values by fitting a truncated lognormal distribution using only values above the LOQ [52]. Concentrations of urinary biomarkers (OH-PAHs, 8-oxodG) were normalized for urinary specific gravity (SG) to reflect urinary dilution. The specific gravity adjustment was calculated according to the formula CONC_SG = CONC * ((1.024 – 1) / (SG – 1)), with CONC_SG as the normalized biomarker concentration, CONC as the measured biomarker concentration per liter urine, and SG as the specific gravity of the urine sample [53].

In addition, we calculated a sum parameter of all measured OH-PAHs (ΣOH-PAHs), which may serve as a proxy for total exposure to the measured OH-PAHs [54]. First, concentrations (µg/L) were converted to molar concentrations (µmol/L) to standardize across differential molecular weights of OH-PAHs. Next, the molar concentrations of all measured OH-PAHs were summed (ΣOH-PAHs, µmol/L) and adjusted for urinary density. All measured biomarkers were natural logarithm (ln) transformed to reduce skewness of distributions. Descriptive statistics provide an overview of study population characteristics, biomarkers of internal exposure to PAHs (OH-PAHs), and effect biomarkers (HCC, leukocyte counts and NLR, 8-oxodG). Biomarker values are presented as geometric means with their 95% confidence interval (95% CI), together with the 25th and 75th percentile. Statistical differences in population characteristics and biomarker levels were assessed, using an independent samples *t*-test for continuous variables and a chi-square test for categorical variables. Pearson’s correlations were calculated between all measured biomarkers. Biomarkers were related to population characteristics in univariate analysis.

Multiple linear regression models were constructed with each individual OH-PAH as an independent variable and each effect biomarker as a dependent variable. We specified three models with different levels of adjustment for a set of a priori defined potential confounders, based on literature [17,30,45,55,56,57]. Model I was adjusted for sex and age. Model II was adjusted for age, sex, BMI, household socio-economic status and season of sampling. Model III was additionally adjusted for smoking and residential exposure to environmental tobacco smoke (ETS) to explore the influence of tobacco smoke, a known source of PAHs and other potential immunotoxic compounds (e.g., heavy metals) on the association. For models with 8-oxodG as an outcome, SG was included as an independent variable in all models as previously recommended [58], to account for variation in dilution in spot urine samples. We considered Model III as our main model.

All assumptions for linear regression analysis were checked. The statistically significant level for associations was set at *p* ≤ 0.05 (two-sided). To quantify associations, the estimated factor change in the outcome variable (β) is presented with a 95% confidence interval (95% CI) for a doubling in OH-PAH concentration. As previously recommended [59], we investigated effect modification by sex by including an interaction term of exposure (OH-PAH) and sex in Model III. The statistically significant level of interactions was set at *p* ≤ 0.20 (two-sided).

To check the robustness of our findings, we additionally adjusted our main models for neighborhood socio-economic status, using the area deprivation index (ADI). Studies suggest that neighborhood SES may influence health outcomes independent of personal SES [60]. Further, leucocyte counts and oxidative stress may be influenced by recent events, including short-term fluctuations in temperature. We additionally adjusted main models for these outcomes with a 2-day mean temperature prior to sampling. Last, infections or illnesses may influence leucocyte counts [57]. We adjusted our leucocyte models for recent health complaints.

As a secondary analysis, the effect of HCC on the relationship of OH-PAHs with leucocyte counts, NLR, and 8-oxodG was assessed for significant associations by adding HCC into each linear regression model and identifying changes in the relationship of OH-PAHs with leucocyte counts, NLR, and 8-oxodG. This is because the effect of OH-PAHs on inflammation and oxidative stress might be directly or indirectly through endocrine stress. Mediation models were constructed, using the Process-macro of Hayes [61] with HCC as a mediator, if the following criteria were fulfilled: (1) the OH-PAH was significantly associated with one of the outcomes, i.e., leukocyte counts, NLR, or 8-oxodG; (2) the OH-PAH was significantly associated with HCC; (3) HCC was significantly associated with the outcome of interest.

## 3. Results

### 3.1. Study Population Characteristics

Table 1 provides an overview of study population characteristics. A total of 393 adolescents (mean age 14.8 ± standard deviation of 0.5 years, 46.6% male) were included in this study. Almost one third of the parents (29% for boys, 27.6% for girls) reported finding it difficult to make ends meet. The average Area Deprivation Index (ADI) of 12.0% in our study population is slightly lower than the overall Flemish ADI of 14.5% [62]. A map geographically illustrating the ADI in Flanders at the municipal level (2017) is presented in Supplementary Materials Figure S1. BMI was normal for 77.6% of boys and 68.1% of girls, in line with the percentage of Flemish boys and girls (10–17 years) with a normal BMI (77.6% and 69.0%, respectively) [63]. Smoking was reported by 4.6% of adolescents. Almost one out of three participants reported health complaints in the fortnight prior to sampling. None of the participants reported the use of systemic glucocorticoid medication. The mean ambient temperature in the 2 days before sampling was 9.2 ± 6.9 °C; no samples were collected during the summer season because of school holidays. We observed no statistical differences in population characteristics, except for the season of sampling and the 2-day average temperature prior to sampling. A larger proportion of girls were recruited during fall and winter (66.7%) compared to boys (39.9%); more girls were recruited at a 2-day average outdoor temperature below 6 °C (44.8% versus 27.9%, respectively).

Table 2 describes the distribution of exposure and effect biomarkers in this study population. The highest individual metabolite concentration was observed for the naphthalene metabolite 2-OHNa. We observed significantly lower geometric mean concentrations of 2-OHNa in boys compared to girls (*p* = 0.002, 3.48 (95% CI: 3.07, 3.95) µg/L and 4.60 (95% CI: 4.08, 5.18) µg/L, respectively) and oftotal ΣOH-PAHs (*p* = 0.004, 0.028 (95% CI: 0.025, 0.032) µmol/L and 0.036 (95% CI: 0.032, 0.040) µmol/L, respectively). Geometric mean concentrations of other measured OH-PAHs did not significantly differ by sex (*p* = 0.743 for 2,3-OHFl, *p* = 0.060 for 2-OHPH, *p* = 0.056 for 3-OHPH, *p* = 0.252 for 1.9-OHPH, and *p* = 0.342 for 1-OHPy). As presented in Table S1, we observed significant correlations among all OH-PAHs. Correlations were the strongest between 3- and 4-ringed OH-PAHs (Pearson’s *r* > 0.50); the 2-ringed 2-OHNa correlated less strongly with 3- and 4-ringed OH-PAHs (Pearson’s *r* < 0.30). We found a strong correlation between ΣOH-PAHs and 2-OHNa (Pearson’s *r* = 0.99), the main contributor to the sum. Table S2 describes the detection frequency of OH-PAHs in the FLEHS-4 study. The phenanthrene metabolite 4-OHPH was detected 6.9% of participants and was not included in further analysis. The detection frequency of the other OH-PAHs ranged between 97.6% for 1-OHPy and 100% for 2-OHNa.

Leucocyte and neutrophil counts were significantly higher in girls compared to boys (*p* = 0.002, 6991 (95% CI: 6775, 7215) cells/µL versus 6471 (95% CI: 6236, 6715) cells/µL and *p* = 0.002, 3870 (95% CI: 3682, 4068) cells/µL versus 3186 (95% CI: 3016, 3366) cells/µL, respectively). NLR was also significantly higher in girls (*p* < 0.001, 1.75 (95% CI: 1.65, 1.86) versus 1.39 (95% CI: 1.31, 1.47)). Geometric mean HCC was 2.85 (95% CI: 2.55, 3.19) pg/mg hair for boys and 3.26 (95% CI:2.94, 3.62) pg/mg hair for girls, with no statistical difference by sex (*p* = 0.083). Further, geometric mean urinary 8-oxodG concentration did not significantly differ between boys and girls (16.80 (95% CI: 15.85, 17.80) µg/L and 17.00 (95% CI: 15.95, 18.10) µg/L, respectively, *p* = 0.790). As shown in Table S1, we observed weak positive correlations of HCC with neutrophil count (Pearson’s *r* = 0.12) and NLR (Pearson’s *r* = 0.15). The urinary 8-oxodG concentration was not significantly correlated with HCC, NLR, or leucocyte counts. Univariate analyses of OH-PAHs and effect biomarker levels in relation to population characteristic and effect biomarkers are presented in Table S3 and Table S4.

### 3.2. Associations between OH-PAHs and Effect Markers

**Figure 1:**
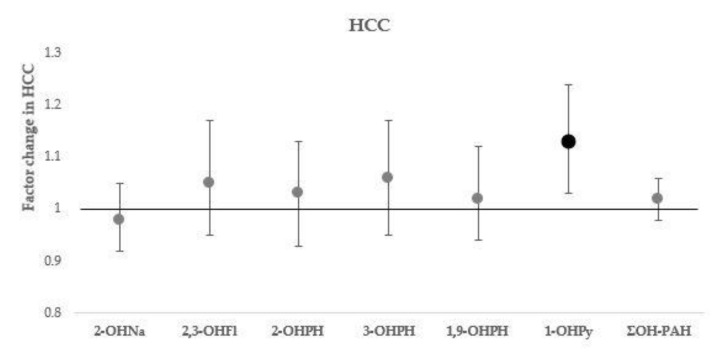
Associations of urinary OH-PAHs concentration with HCC. Effect estimates are presented with their 95% confidence interval (95% CI) as the factor change in HCC for a doubling in OH-PAH concentration. Models adjusted for sex, age, BMI, household socio-economic status, season of sampling, smoking, and residential exposure to environmental tobacco smoke. Significant associations (*p*-value ≤ 0.05) are marked in bold. Abbreviations: OH-PAHs—hydroxylated polycyclic aromatic hydrocarbon; 1-OHPy—1-hydroxypyrene; 2-OHNa—2-hydroxynaphthalene; 2,3-OHFl—sum of 2-hydroxyfluorene and 3-hydroxyfluorene; 2-OHPH—2-hydroxyphenanthrene; 3-OHPH—3-hydroxyphenanthrene; 1,9-OHPH–sum of 1-hydroxyphenanthrene and 9-hydroxyphenanthrene; ΣOH-PAH—sum of molar concentrations of all measured OH-PAH; HCC—hair cortisol concentration.

**Figure 2:**
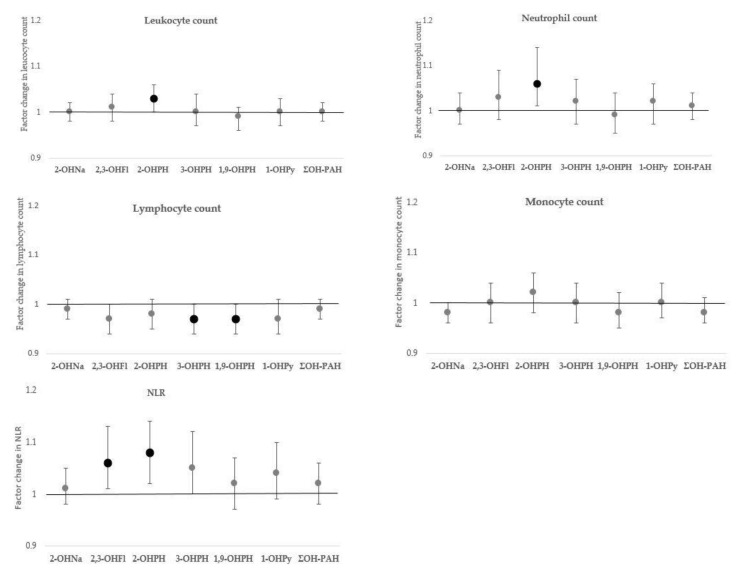
Associations of urinary OH-PAHs concentration with leucocyte counts and NLR in serum. Effect estimates are presented with their 95% confidence interval (95% CI) as the factor change in leucocyte count or NLR for a doubling in OH-PAH concentration. Models adjusted for sex, age, BMI, household socio-economic status, season of sampling, smoking, and residential exposure to environmental tobacco smoke. Significant associations (*p*-value ≤ 0.05) are marked in bold. Abbreviations: OH-PAHs—hydroxylated polycyclic aromatic hydrocarbon; 1-OHPy—1-hydroxypyrene; 2-OHNa—2-hydroxynaphthalene; 2,3-OHFl—sum of 2-hydroxyfluorene and 3-hydroxyfluorene; 2-OHPH—2-hydroxyphenanthrene; 3-OHPH—3-hydroxyphenanthrene; 1,9-OHPH—sum of 1-hydroxyphenanthrene and 9-hydroxyphenanthrene; ΣOH-PAH—sum of molar concentrations of all measured OH-PAHs; NLR—neutrophil–lymphocyte ratio.

**Figure 3:**
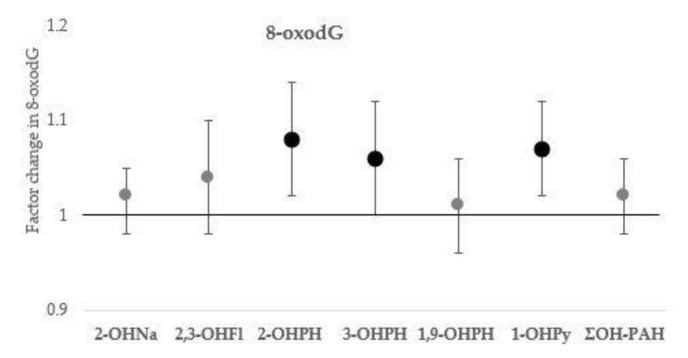
Associations of urinary OH-PAHs concentration with urinary 8-oxodG. Effect estimates are presented with their 95% confidence interval (95% CI) as the factor change in 8-oxodG for a doubling in OH-PAH concentration. Models adjusted for urinary specific gravity, sex, age, BMI, household socio-economic status, season of sampling, smoking, and residential exposure to environmental tobacco smoke. Significant associations (*p*-value ≤ 0.05) are marked in bold. Abbreviations: OH-PAHs—hydroxylated polycyclic aromatic hydrocarbon; 1-OHPy—1-hydroxypyrene; 2-OHNa—2-hydroxynaphthalene; 2,3-OHFl—sum of 2-hydroxyfluorene and 3-hydroxyfluorene; 2-OHPH—2-hydroxyphenanthrene; 3-OHPH—3-hydroxyphenanthrene; 1,9-OHPH—sum of 1-hydroxyphenanthrene and 9-hydroxyphenanthrene; ΣOH-PAH—sum of molar concentrations of all measured OH-PAHs; 8-oxodG—8-oxo-7,8-dihydro-2’-deoxyguanosine.

The associations of urinary OH-PAHs with HCC, leucocyte counts, and NLR, and 8-oxodG are presented with increasing level of adjustment in Table 3, Table 4 and Table 5; estimates of the associations in fully adjusted models (model III) are presented in Figure 1, Figure 2 and Figure 3.

The urinary 1-OHPy concentration was significantly associated with HCC. As illustrated in Figure 1, an increase in HCC with a factor of 1.13 (95% CI: 1.03, 1.24, *p* = 0.013) was estimated for a doubling in urinary 1-OHPy concentration. We did not observe significant associations between other measured urinary OH-PAHs or ΣOH-PAH and HCC.

As shown in Figure 2, we observed a significant association of urinary 2-3-OHFl concentration with NLR. For a doubling in urinary 2-3-OHFl concentration, an increase in NLR with a factor of 1.06 (95% CI: 1.01, 1.13, *p* = 0.037) was estimated. The urinary concentration of 2-OHPH was significantly associated with total leucocyte count, neutrophil count, and NLR. A doubling in 2-OHPH concentration was associated with an increase in total leucocyte count with a factor of 1.03 (95% CI: 1.00, 1.06), *p* = 0.048), an increase in neutrophil count with a factor of 1.06 (95% CI: 1.01, 1.11, *p* = 0.024), and an increase in NLR with a factor of 1.08 (95% CI: 1.02, 1.14, *p* = 0.007). A doubling in 3-OHPH was associated with a decrease in lymphocyte counts with a factor of 0.97 (95% CI: 0.94, 1.00, *p* = 0.050); the association of 3-OHPH with NLR was borderline significant (β = 1.05 (95% CI: 1.00, 1.12), *p* = 0.066). Urinary 1,9-OHPH concentration was inversely associated with lymphocyte count (β = 0.97 (95% CI: 0.94, 1.00), *p* = 0.040).

As illustrated in Figure 3, urinary 2-OHPH, 3-OHPH, and 1-OHPy were significantly associated with urinary 8-oxodG concentration. A doubling in urinary 1-OHPy concentration was associated with an increase in 8-oxodG with a factor of 1.07 (95% CI: 1.02, 1.13, *p* = 0.007); a doubling in 2-OHPH and 3-OHPH concentration was associated with an increase in 8-oxodG with a factor of 1.08 (95% CI: 1.02, 1.14, *p* = 0.005) and a factor of 1.06 (95% CI: 1.00, 1.12, *p* = 0.045), respectively. We did not observe significant associations of the ΣOH-PAHs with leukocyte counts or 8-oxodG.

Heterogeneity of results by sex (*p*-value of the interaction term) is presented in Table S5; effect estimates of associations that significantly differed by sex are presented for boys and girls in Table S6. We observed significant effect modification by sex for HCC in relation to 2-OHNa (*p*-interaction = 0.067), 2,3-OHFl (*p*-interaction = 0.154), and 2-OHPH (*p*-interaction = 0.079). The interaction models, however, revealed no significant associations of the aforementioned OH-PAHs with HCC in boys nor girls. Furthermore, we observed significant heterogeneity of results by sex for the association of 2,3-OHFl with lymphocyte count and NLR (*p*-interaction = 0.012 and *p*-interaction = 0.147, respectively). In girls, a doubling in urinary 2,3-OHFl concentration was associated with a significant decrease in lymphocyte count with a factor of 0.94 (95% CI: 0.90, 0.98, *p* = 0.007); no significant change in lymphocyte count was observed in boys (β = 1.02, 95% CI: 0.97, 1.07, *p* = 0.384). The analysis also revealed that the significant association of 2-3-OHFl with NLR was driven by girls. A doubling in 2,3-OHFl concentration was associated with a significant increase in girls’ NLR with a factor of 1.10 (95% CI: 1.02, 1.18, *p* = 0.012). In boys, no significant changes in NLR were found in relation to urinary 2,3-OHFl concentration (β = 1.02, 95% CI: 0.94, 1.10, *p* = 0.710). Associations of OH-PAHs with 8-oxodG did not significantly differ by sex.

For all outcomes, results remained robust after additional adjustment for neighborhood SES (Table S7). Further, additional adjustment for 2-day mean temperature of associations between OH-PAHs and leucocyte counts NLR and 8-oxodG was of little influence on the strength and direction of associations (Table S8). Adjustment of our leucocyte models for recent health complaints (Table S9) was of little to no influence on our main results.

In a secondary analysis, we explored whether HCC was associated with leucocyte counts, NLR, and 8-oxodG and whether HCC was of influence on associations of OH-PAHs with these outcomes (Table S10). We observed a significant association of HCC with NLR. The NLR increased with a factor of 1.05 (95% CI: 1.01, 1.09, *p* = 0.023) for a doubling in HCC; the association did not significantly differ by sex (*p*-interaction = 0.443). We observed no significant associations of HCC with 8-oxodG or leucocyte counts. Additional adjustment of our leucocyte, NLR and 8-oxodG models with HCC was of little influence on the strength of the observed associations. In particular, 2,3-OHFl and HCC were independently associated with NLR (β = 1.06 (95% CI: 1.00, 1.12), *p* = 0.050 for 2,3-OHFl and β = 1.04 (95% CI: 1.00, 1.09), *p* = 0.030 for HCC). Furthermore, we observed significant associations of both 2-OHPH and HCC with NLR (β = 1.08 (95% CI: 1.02, 1.14), *p* = 0.011 and β = 1.05 (95% CI: 1.01, 1.09), *p* = 0.027 respectively). The criteria for mediation analysis were not met for any of the observed associations.

## 4. Discussion

Our results indicate that exposure to PAHs may be associated with oxidative stress, inflammation, and chronic endocrine stress in a general population of adolescents. We evaluated all associations with different levels of adjustment for potential confounders. All results were robust to control for demographic (age, sex, and household SES), anthropometric (BMI), lifestyle (smoking and exposure to ETS) and meteorological factors (season and temperature). All potential confounders have previously been suggested to relate to exposure to PAHs [4,38,55,64], to inflammation [38,57,65], oxidative and endocrine stress [17,30]. We found no indication of an influence of neighborhood SES on the observed associations.

In line with the literature, we observed significant associations of OH-PAHs with the oxidative stress biomarker 8-oxodG. These findings may be relevant to public health, as oxidative damage to nucleic acids is a well-known initiator in the pathogenesis of cardiovascular diseases, neurodegenerative disorders, and cancer [15]. Previous studies have shown that an increase in 8-oxodG levels in urine is most probably detected if oxidative stress is not limited to a specific organ or tissue, but it occurs systemically [17]. PAH may augment the generation of Reactive Oxygen Species (ROS) through their enzymatic transformation in the liver that leads to the formation of reactive intermediates, which may react with DNA and proteins and increase levels of ROS [13]. Furthermore, PAHs and PAH metabolites are ligands for aryl hydrocarbon receptors, and binding to these receptors may also result in oxidative stress [15]. Urinary 1-OHPy levels have previously been associated with 8-oxodG in a pooled analysis, including 2283 Flemish adolescents (14–18 years) from nine different cross-sectional FLEHS surveys in the period 1999–2018 [17]. A doubling in urinary 1-OHPy concentrations was associated with a factor of 1.05 (95% CI: 1.02, 1.08) increase in 8-oxodG, in line with the results in this study. In previous FLEHS-studies, 1-OHPy was the only OH-PAH measured. Urinary 1-OHPy levels have also been associated with oxidative stress biomarkers in European children [14].

In addition, we observed significant associations of the phenanthrene metabolite 2-OHPH with leukocyte count, neutrophil count, and NLR, which are immune biomarkers of inflammation. The sum of fluorene metabolites (2,3-OHFl) was also associated with NLR, with stronger associations observed in girls compared to boys. Differences in associations by sex between fluorene metabolites and inflammatory biomarkers have recently also been observed in the American National Health and Nutrition Examination Survey (NHANES, data from 2003−2016) [38]. In this study, including 3194 adolescents (12–19 years), urinary concentrations of 2-hydroxy-fluorene were significantly associated with C-reactive protein and leucocyte count in girls but not in boys. Possible explanations may be that girls have higher levels of activity of CYP1A1 enzymes, and PAH may produce higher toxicity through CYP1A1 metabolic activation [38]. Another reason for differential results with regard to fluorene metabolites may lie in the estrogenic activity that has been reported for fluorene [64]. Estrogen activity may modulate the differentiation, maturation, lifespan, and effector functions of innate immune cells, including neutrophils, and literature has shown differential immune responses in females and males [65]. In our study, girls exhibited significantly higher levels of neutrophils and NLR compared to boys. In a previous NHANES study (*n* = 660, 12–19 years), a significant association of 2-OHPH was observed with serum C-reactive protein levels in females only, whereas an increase in 9-hydroxyfluorene was related to an increase in CRP in males only [13]. In adult participants of the NHANES study (*n* = 2488), significant associations of the sum of urinary PAH metabolites (naphthalene, fluorene, and phenanthrene) with leukocyte count and CRP were more evident among men than women [66]. These studies indicate that the strength of associations between OH-PAHs and inflammatory biomarkers may differ by the individual properties of each OH-PAH and by age. The exact mechanisms that drive these sex-dependent differences remain incompletely characterized. To the best of our knowledge, associations of urinary OH-PAHs with neutrophil–lymphocyte ratio (NLR) have not been reported previously. Our findings may be relevant to adolescents’ health. Systemic inflammation and immune dysregulation, characterized by an increased neutrophil–lymphocyte ratio (NLR), may increase susceptibility to infection and cancer [67]. Moreover, persistent systemic inflammation may promote the development of a series of chronic diseases, such as cardiovascular and cognitive diseases [2].

This study is the first to observe a significant association between urinary 1-OHPy and HCC as a chronic endocrine stress biomarker. Research on GC homeostasis in relation to exposure to EDCs has gained more attention recently [19]; however, the link between exposure to PAHs and GC homeostasis remains an understudied research area. Several pathways by which PAHs may affect GC homeostasis have been postulated. PAHs have been classified as endocrine-disrupting chemicals (EDCs) [1,20]. EDCs may modulate the enzymes involved in the production, transformation, or elimination of GC hormones and may alter the absolute and relative concentrations of hormones in blood tissues [68,69]. Further, PAH-induced systemic oxidative stress may lead to inflammation and hypothalamic–pituitary–adrenal axis (HPA) axis activation, which may result in increased cortisol secretion [70]. Moreover, due to their lipophilicity, PAHs may cross the blood–brain barrier to directly interact with brain tissues, including the hypothalamus [71]. Experimental animal research used concentrated urban particles (EHC-93) from Ottawa, Canada, in which PAHs are known to be present to study the impact on GC homeostasis [72]. Exposure of healthy rodents to EHC-93 increased plasma levels of glucocorticoid (GC) hormones, confirming the activation of the hypothalamic-pituitary-adrenal axis [73]. However, EHC-93 is a chemical mixture that also includes other potential EDCs such as heavy metals. Further experimental and epidemiological research is needed to confirm our results.

Maintenance of GC homeostasis is essential for the appropriate functioning of many cell types, including immune cells [20]. In our secondary analysis, we observed a significant association HCC with NLR, suggesting a significant impact of 3-month cumulative cortisol secretion on inflammation and immune functioning in adolescents. We observed no indication for an intermediate role of HCC in the association between PAH exposure and NLR. HCC has previously been associated with an increased NLR in German adults [57]. Our findings regarding chronic endocrine stress in relation to 1-OHPy levels, if confirmed in future studies, may be relevant to adolescents’ health. Increases in HCC in children and adolescents have been linked to a higher BMI, behavioral problems, and worse cognitive performance [58,59,60]. Moreover, chronically elevated cortisol levels during adolescence may lead to adverse health effects that persist into adulthood, including anxiety, cognitive dysfunction, metabolic disorders, and cardiovascular disease [22,27].

Our study has several strengths. Urinary concentrations of PAH metabolites (OH-PAHs) reflect the integrated internal exposure through different routes of PAHs exposure, i.e., inhalation, ingestion, and dermal uptake [74]. In FLEHS-4, we recruited a representative sample of Flemish adolescents (14–15 years), a population subgroup with increased vulnerability to environmental exposures, whereas most PAH-related studies have focused on adults. Samples were collected by a small team of trained nurses, following a strict protocol to ensure low variability in the sampling method across our study population. We simultaneously assessed endocrine stress, inflammation, and oxidative stress to gain more insight into the relation between these biological pathways. We measured metabolites of four different PAHs, whereas many studies use 1-OHPy or summed OH-PAHs as biomarkers of exposure to the mixture of PAHs. Measuring different PAHs and several metabolites per PAH allows to identify variations in mechanisms and potency across PAHs [13].

In this study, we did not observe significant associations between the sum of measured OH-PAHs and effect biomarkers. However, we found metabolites of different PAHs to be differentially associated with the studied biological mechanisms and observed differences in the strength of associations among metabolites of phenanthrene. Measuring different OH-PAHS may also provide more insight into PAH exposure. The OH-PAHs in this study all correlated to some degree, with correlations between three- and four-ringed fluorene, phenanthrene, and pyrene metabolites being stronger than correlations between these metabolites and the two-ringed naphthalene metabolite. Generally, the main source of exposure to naphthalene is through inhalation (mostly ambient pollution), and the sources of exposure to larger PAHs are more diverse (diet and inhalation) [75]. Although food is considered an important source of PAH exposure to larger PAHs, part of this contamination may arise from air pollution with PAHs [76]. Flanders is one of the European air pollution hot spots [77], and heating of buildings and vehicular emissions are the major sources of PAHs emissions in ambient air [78].

Some limitations of the study also need to be addressed. PAHs that may co-vary with those assessed here but were not measured could have biologically relevant effects on our outcomes [79]. In this study, we investigated a limited set of pathophysiological mechanisms in relation to PAH exposure. A more integrated view of health effects in relation to PAH exposure would be an added value. The Toxic Equivalency Factor (TEF) provides an estimate of the carcinogenic potency of a PAH relative to benzo[a]pyrene (BaP), the reference standard [80,81]. It is derived from animal experiments and in vitro assays. All parent compounds of the OH-PAHs we determined in this study (naphthalene, fluorene, phenanthrene, and pyrene) were classified with a TEF of 0.001 [81]. However, equivalency factors may not necessarily apply to the endpoints we measured in this study. Extending the concept of TEF to different modes of action could provide a better insight into PAH-associated health effects [82]. Our exposure measurement was limited to a single spot urine sample. PAHs are rapidly metabolized and have relatively short half-lives [1]. However, PAHs are ubiquitous in the environment, exposure to PAHs occurs continuously, and previous studies have shown a single urine sample to be representative of an individual’s normal PAH exposure level [13,83]. Alterations in urinary OH-PAHs levels in response to exposure requires several hours to days; this may give rise to uncertainty whether measured OH-PAHs levels are causally related to the effect of biomarker levels. A longitudinal study with multiple measurements over time of both OH-PAHs and effect biomarkers, and assessing changes in those measurements, would provide a more accurate estimate of associations between OH-PAHs and effect biomarkers. However, we assume that at population level, PAH exposure through air and food intake is relatively constant as it is linked to residence and lifestyle habits. The use of exposure biomarkers has the advantage that it reflects more precisely the actual integrated exposure from different exposure pathways compared to modeled intake data from food questionnaires or long-term air pollution exposure models. The phenanthrene metabolite 4-OHPH was detected in a small proportion of participants. The LOQ for 4-OHPH in our study was 0.014 µg/L, in line with the LOQ of the other measured phenanthrene metabolites. However, urinary levels of 4-OHPH are in general lower than levels of the other phenanthrene metabolites [4,84]. The recent German human biomonitoring study (GerES V, 2014–2017) measured 4-OHPH levels using a method with an LOQ of 0.001 µg/L and reported median 4-OHPH values of 0.04 µg/L, which is below our LOQ. [4]. In future research, striving towards a lower LOQ for 4-OHPH could enable the detection of 4-OHPH in the general population. We measured leucocyte counts at one time point; leucocytes in peripheral blood have a lifespan ranging from hours to days. A low level of intraindividual variation in leucocyte count over time was established in a 6.5-year longitudinal study in healthy men [85]. Results in this study underpinned the utility of a single measurement of leucocyte count as a biomarker of immune responses in epidemiologic studies. Associations of leucocyte counts with long-term environmental exposures have since been evaluated in a multitude of epidemiological studies [39,86,87,88]. We adjusted associations between OH-PAHs and outcomes for a priori defined potential confounders. However, we cannot exclude residual confounding by other environmental and/or social stressors. Assessment of smoking and residential exposure to ETS was based on self-reported questionnaire data; measuring cotinine levels as an indicator of tobacco exposure would be an added value in future studies. We did not investigate possible routes of exposure to PAHs other than smoking. We performed a cross-sectional study, which limited us in our ability to establish causality. Additional longitudinal research is necessary to clarify the consequences of these PAHs-associated early pathophysiological changes on adolescents’ health.

## 5. Conclusions

This study indicates that higher urinary OH-PAHs concentrations are simultaneously but differently associated with increased levels of chronic endocrine stress, oxidative stress, and inflammation in a general population of Flemish adolescents. These findings may contribute to a better understanding of the pathways through which PAHs induce adverse health effects in adolescents.

## Figures and Tables

**Table 1:** Demographic study population characteristics.

Characteristics	All (*n* = 393)	%	Boys (*n* = 183)	%	Girls (*n* = 210)	%	*p*-Value
Age (years)							0.855
<14.5	103	26.2	46	25.1	57	27.1	
14.5−15.5	258	65.6	121	66.1	137	65.2	
>15.5	32	8.1	16	8.7	16	7.6	
Perceived income adequacy							0.668
Difficult	111	28.2	53	29	58	27.6	
Rather easy	125	31.8	61	33.3	64	30.5	
Easy–very easy	151	38.4	66	36.1	85	40.5	
Missing	6	1.5	3	1.6	3	1.4	
Area Deprivation Index							0.180
0−5.3%	94	23.9	40	21.9	54	25.7	
5.4−9.3%	99	25.2	41	22.4	58	27.6	
9.4−15.5%	101	25.7	47	25.6	54	25.7	
>15.5%	99	25.2	55	30.1	44	21	
Body Mass Index ^a^							0.053
Underweight	32	8.1	15	8.2	17	8.1	
Normal weight	285	72.5	142	77.6	143	68.1	
Overweight, obese	76	19.3	26	14.2	50	23.8	
Smoking							0.427
No	374	95.2	172	94	202	96.2	
Yes	18	4.6	10	5.5	8	3.8	
Missing	1	0.3	1	0.5	0	0	
Residential exposure to ETS							0.809
No	351	89.3	165	90.2	186	88.6	
Yes	40	10.2	18	9.8	22	10.5	
Missing	2	0.5	0	0	2	1	
Recent health complaints							0.297
No	277	70.5	134	73.2	143	68.1	
Yes	115	29.3	49	26.8	66	31.4	
Missing	1	0.3	0	0	1	0.5	
Season of sampling							**0.001**
Winter	123	31.3	48	26.2	75	35.7	
Spring	180	45.8	110	60.1	70	33.3	
Summer	-	-	-	-	-	-	
Fall	90	22.9	25	13.7	65	31	
2-day mean temperature (°C)							**0.001**
<6	145	36.9	51	27.9	94	44.8	
6−12	137	34.9	58	31.7	79	37.6	
>12	111	28.2	74	40.4	37	17.6	

Data in number and percentages (%), ^a^ BMI classes based on age- and sex-specific Belgian growth curves. Significant differences in characteristics by sex (*p*-value ≤ 0.05) are marked in bold. Abbreviations: ETS—environmental tobacco smoke.

**Table 2:** Distribution of exposure and effects biomarker.

Biomarker	All (*n* = 393)			Boys (*n* = 183)	Girls (*n* = 210)	
	GM (95% CI)	p25	p75	GM (95% CI)	GM (95% CI)	*p*-Value
**OH-PAHs (µg/L) ^a^**						
2-OHNa *	4.04 (3.70, 4.41)	2.15	7.07	3.48 (3.07, 3.95)	4.60 (4.08, 5.18)	**0.002**
2,3-OHFl *	0.209 (0.197, 0.221)	0.144	0.277	0.207 (0.190, 0.224)	0.211 (0.195, 0.228)	0.743
2-OHPH	0.074 (0.069, 0.078)	0.051	0.101	0.078 (0.072, 0.085)	0.070 (0.065, 0.076)	0.06
3-OHPH	0.073 (0.069, 0.078)	0.052	0.099	0.077 (0.071, 0.084)	0.070 (0.065, 0.075)	0.056
1,9-OHPH	0.111 (0.104, 0.118)	0.074	0.149	0.107 (0.098, 0.116)	0.115 (0.105, 0.126)	0.252
1-OHPy **	0.067 (0.063, 0.074)	0.048	0.096	0.065 (0.060, 0.071)	0.069 (0.063, 0.074)	0.342
ΣOH-PAH (µmol/L) ^a,^**	0.032 (0.029, 0.035)	0.018	0.052	0.028 (0.025, 0.032)	0.036 (0.032, 0.040)	**0.004**
**Effect biomarkers**						
HCC (pg/mg hair)	3.07 (2.84, 3.31)	2.09	4.38	2.85 (2.55, 3.19)	3.26 (2.94, 3.62)	0.083
Leucocytes (cells/µL)	6744 (6583, 6910)	5675	7925	6471 (6236, 6715)	6991 (6775, 7215)	**0.002**
Neutrophils (cells/µL)	3535 (3403, 3672)	2757	4636	3186 (3016, 3366)	3870 (3682, 4068)	**0.002**
Lymphocytes (cells/µL)	2250 (2195, 2307)	1941	2627	2297 (2216, 2381)	2210 (2136, 2287)	0.124
Monocytes (cells/µL)	562 (546, 579)	458	692	570 (547, 594)	555 (533, 578)	0.373
NLR	1.57 (1.50, 1.64)	1.19	2.1	1.39 (1.31, 1.47)	1.75 (1.65, 1.86)	**<0.001**
8-oxodG (µg/L) ^a^	16.90 (16.19, 17.65)	13.41	21.43	16.80 (15.85, 17.80)	17.00 (15.95, 18.10)	0.790

^a^ Specific gravity normalized concentrations. * Data available for 392 participants. ** Data available for 391 participants. Significant differences in biomarker levels by sex (*p*-value ≤ 0.05) are marked in bold. Abbreviations: OH-PAHs—hydroxylated polycyclic aromatic hydrocarbon; 2-OHNa—2-hydroxy-naphthalene; 2,3-OHFl—sum of 2-hydroxy-fluorene and 3-hydroxy-fluorene; 2-OHPH—2-hydroxy-phenanthrene; 3-OHPH—3-hydroxy-phenanthrene; 1,9-OHPH—sum of 1-hydroxy-phenanthrene and 9-hydroxy-phenanthrene; 1-OHPy—1-hydroxy-pyrene; ΣOH-PAH—sum of molar concentrations of all measured OH-PAHs; HCC—hair cortisol concentration; NLR—neutrophil–lymphocyte ratio; 8-oxodG—8-oxo-7,8-dihydro-2’-deoxyguanosine.

**Table 3:** Linear regression analyses of urinary OH-PAHs concentrations with hair cortisol concentration.

OH-PAHs	Model I		Model II		Model III	
	*n*	β (95% CI)	*n*	β (95% CI)	*n*	β (95% CI)
2-OHNa	392	1.00 (0.94, 1.06)	386	0.98 (0.92, 1.05)	384	0.98 (0.92, 1.05)
2,3-OHFl	392	1.08 (0.98, 1.18)	386	1.05 (0.95, 1.15)	384	1.05 (0.95, 1.17)
2-OHPH	393	1.05 (0.96, 1.15)	387	1.01 (0.92, 1.12)	385	1.03 (0.93, 1.13)
3-OHPH	393	1.06 (0.96, 1.17)	387	1.05 (0.95, 1.16)	385	1.06 (0.95, 1.17)
1,9-OHPH	393	1.03 (0.95, 1.12)	387	1.01 (0.93, 1.10)	385	1.02 (0.94, 1.12)
1-OHPy	391	**1.12 (1.02, 1.23)**	385	**1.12 (1.02, 1.23)**	383	**1.13 (1.03, 1.24)**
ΣOH-PAH	391	1.02 (0.98, 1.06)	385	1.02 (0.98, 1.06)	383	1.02 (0.98, 1.06)

Effect estimates β are presented with their 95% confidence interval (95% CI) as the factor change in HCC for a doubling in OH-PAH concentration. Significant associations (*p*-value ≤ 0.05) are marked in bold. Model I adjusted for sex and age; Model II adjusted for sex, age, BMI, household socio-economic status, and season of sampling; Model III adjusted for sex, age, BMI, household socio-economic status, season of sampling, smoking, and residential exposure to environmental tobacco smoke. Abbreviations: OH-PAHs—hydroxylated polycyclic aromatic hydrocarbon; 2-OHNa—2-hydroxynaphthalene; 2,3-OHFl—sum of 2-hydroxyfluorene and 3-hydroxyfluorene; 2-OHPH—2-hydroxyphenanthrene; 3-OHPH—3-hydroxyphenanthrene; 1,9-OHPH—sum of 1-hydroxyphenanthrene and 9-hydroxyphenanthrene; 1-OHPy—1-hydroxypyrene; ΣOH-PAH—sum of molar concentrations of all measured OH-PAHs; HCC—hair cortisol concentration.

**Table 4:** Linear regression analyses of urinary OH-PAHs concentrations with leukocyte counts and NLR.

OH-PAHs	Model I		Model II		Model III	
	*n*	β (95% CI)	*n*	β (95% CI)	*n*	β (95% CI)
**Leukocytes**						
2-OHNa	392	1.00 (0.98, 1.02)	386	1.00 (0.98, 1.02)	384	1.00 (0.98, 1.02)
2,3-OHFl	392	1.02 (0.99, 1.05)	386	1.01 (0.98, 1.05)	384	1.01 (0.98, 1.04)
2-OHPH	393	**1.03 (1.00 1.07)**	387	**1.03 (1.00, 1.07)**	385	**1.03 (1.00, 1.06)**
3-OHPH	393	1.01 (0.98, 1.04)	387	1.01 (0.97, 1.04)	385	1.00 (0.97, 1.04)
1,9-OHPH	393	0.99 (0.96, 1.02)	387	0.99 (0.96, 1.01)	385	0.99 (0.96, 1.01)
1-OHPy	391	1.00 (0.98, 1.03)	385	1.00 (0.97, 1.03)	383	1.00 (0.97, 1.03)
ΣOH-PAH	391	1.00 (0.98, 1.06)	385	1.00 (0.98, 1.02)	383	1.00 (0.98, 1.02)
**Neutrophils**						
2-OHNa	392	1.01 (0.98, 1.04)	386	1.01 (0.98, 1.04)	384	1.00 (0.97, 1.04)
2,3-OHFl	392	1.04 (1.00, 1.09)	386	1.04 (0.99, 1.09)	384	1.03 (0.98, 1.09)
2-OHPH	393	**1.06 (1.02, 1.11)**	387	**1.06 (1.01, 1.11)**	385	**1.06 (1.01, 1.11)**
3-OHPH	393	1.03 (0.98, 1.08)	387	1.02 (0.98, 1.08)	385	1.02 (0.97, 1.07)
1,9-OHPH	393	1.00 (0.96, 1.04)	387	0.99 (0.95, 1.04)	385	0.99 (0.95, 1.04)
1-OHPy	391	1.02 (0.98, 1.07)	385	1.02 (0.97, 1.07)	383	1.02 (0.97, 1.06)
ΣOH-PAH	391	1.01 (0.98, 1.04)	385	1.01 (0.98, 1.04)	383	1.01 (0.98, 1.04)
**Lymphocytes**						
2-OHNa	392	0.99 (0.97, 1.01)	386	0.99 (0.97, 1.01)	384	0.99 (0.97, 1.01)
2,3-OHFl	392	0.98 (0.95, 1.01)	386	0.98 (0.95, 1.01)	384	0.97 (0.94, 1.00)
2-OHPH	393	0.99 (0.96, 1.02)	387	0.98 (0.95, 1.02)	385	0.98 (0.95, 1.01)
3-OHPH	393	0.98 (0.94, 1.01)	387	0.97 (0.94, 1.00)	385	**0.97 (0.94, 1.00)**
1,9-OHPH	393	0.98 (0.95, 1.01)	387	**0.97 (0.94, 1.00)**	385	**0.97 (0.94, 1.00)**
1-OHPy	391	0.98 (0.95, 1.21)	385	0.97 (0.94, 1.00)	383	0.97 (0.94, 1.01)
ΣOH-PAH	391	0.99 (0.97, 1.01)	385	0.99 (0.97, 1.02)	383	0.99 (0.97, 1.01)
**Monocytes**						
2-OHNa	392	0.98 (0.96, 1.01)	386	0.98 (0.96, 1.01)	384	0.98 (0.96, 1.00)
2,3-OHFl	392	1.00 (0.96, 1.03)	386	1.00 (0.96, 1.04)	384	1.00 (0.96, 1.04)
2-OHPH	393	1.02 (0.98, 1.06)	387	1.02 (0.98, 1.06)	385	1.02 (0.98, 1.06)
3-OHPH	393	1.00 (0.97, 1.04)	387	1.00 (0.96, 1.04)	385	1.00 (0.96, 1.04)
1,9-OHPH	393	0.98 (0.95, 1.02)	387	0.98 (0.95, 1.02)	385	0.98 (0.95, 1.02)
1-OHPy	391	1.00 (0.97, 1.04)	385	1.00 (0.97, 1.04)	383	1.00 (0.97, 1.04)
ΣOH-PAH	391	0.98 (0.96, 1.01)	385	0.98 (0.96, 1.01)	383	0.98 (0.96, 1.01)
**Neutrophil–lymphocyte ratio (NLR)**						
2-OHNa	392	1.01 (0.98, 1.05)	386	1.01 (0.98, 1.05)	384	1.01 (0.98, 1.05)
2,3-OHFl	392	**1.06 (1.01, 1.12)**	386	**1.06 (1.01, 1.12)**	384	**1.06 (1.01, 1.13)**
2-OHPH	393	**1.08 (1.02, 1.13)**	387	**1.08 (1.02, 1.14)**	385	**1.08 (1.02, 1.14)**
3-OHPH	393	1.05 (0.99, 1.11)	387	1.05 (1.00, 1.11)	385	1.05 (1.00, 1.12)
1,9-OHPH	393	1.02 (0.97, 1.06)	387	1.02 (0.98, 1.07)	385	1.02 (0.97, 1.07)
1-OHPy	391	1.04 (0.99, 1.10)	385	1.04 (0.99, 1.09)	383	1.04 (0.99, 1.10)
ΣOH-PAH	391	1.02 (0.98, 1.06)	385	1.02 (0.98, 1.06)	383	1.02 (0.98, 1.06)

Effect estimates β are presented with their 95% confidence interval (95% CI) as the factor change in leucocyte count or NLR for a doubling in OH-PAH concentration. Significant associations (*p*-value ≤ 0.05) are marked in bold. Model I is adjusted for sex and age; Model II is adjusted for sex, age, BMI, household socio-economic status, and season of sampling; Model III is adjusted for sex, age, BMI, household socio-economic status, season of sampling, smoking, and residential exposure to environmental tobacco smoke. Abbreviations: OH-PAH—hydroxylated polycyclic aromatic hydrocarbon; 2-OHNa—2-hydroxynaphthalene; 2,3-OHFl—sum of 2-hydroxyfluorene and 3-hydroxyfluorene; 2-OHPH—2-hydroxyphenanthrene; 3-OHPH—3-hydroxyphenanthrene; 1,9-OHPH—sum of 1-hydroxyphenanthrene and 9-hydroxyphenanthrene; 1-OHPy—1-hydroxypyrene; ΣOH-PAHs—sum of molar concentrations of all measured OH-PAH; NLR—neutrophil–lymphocyte ratio.

**Table 5:** Linear regression analyses of urinary OH-PAHs concentrations with urinary 8-oxodG.

OH_PAHs	Model I		Model II		Model III	
	*n*	β (95% CI)	*n*	β (95% CI)	*n*	β (95% CI)
2-OHNa	392	1.02 (0.99, 1.05)	386	1.02 (0.99, 1.05)	384	1.02 (0.98, 1.05)
2,3-OHFl	392	1.04 (0.99, 1.10)	386	1.04 (1.00, 1.11)	384	1.04 (0.98, 1.10)
2-OHPH	393	**1.07 (1.02, 1.12)**	387	**1.08 (1.03, 1.14)**	385	**1.08 (1.02, 1.14)**
3-OHPH	393	1.05 (1.00, 1.11)	387	**1.06 (1.01, 1.12)**	385	**1.06 (1.00, 1.12)**
1,9-OHPH	393	1.00 (0.96, 1.05)	387	1.01 (0.97, 1.06)	385	1.01 (0.96, 1.06)
1-OHPy	391	**1.07 (1.02, 1.12)**	385	**1.08 (1.03, 1.13)**	383	**1.07 (1.02, 1.13)**
ΣOH-PAH	391	1.02 (0.99, 1.06)	385	1.02 (0.99, 1.06)	383	1.02 (0.98, 1.06)

Effect estimates β are presented with their 95% confidence interval (95% CI) as the factor change in 8-oxodG for a doubling in OH-PAH concentration. Significant associations (*p*-value ≤ 0.05) are marked in bold Model I adjusted for urinary specific gravity, sex, and age. Model II adjusted for urinary specific gravity, sex, age, BMI, household socio-economic status, and season of sampling. Model III adjusted for urinary specific gravity, sex, age, BMI, household socio-economic status, season of sampling, smoking, and residential exposure to environmental tobacco smoke. Abbreviations: OH-PAH—hydroxylated polycyclic aromatic hydrocarbon; 1-OHPy—1-hydroxypyrene; 2-OHNa—2-hydroxynaphthalene; 2,3-OHFl—sum of 2-hydroxyfluorene and 3-hydroxyfluorene; 2-OHPH—2-hydroxyphenanthrene; 3-OHPH—3-hydroxyphenanthrene; 1,9-OHPH—sum of 1-hydroxyphenanthrene and 9-hydroxyphenanthrene; ΣOH-PAH—sum of molar concentrations of all measured OH-PAHs; 8-oxodG—8-oxo-7,8-dihydro-2’-deoxyguanosine.

## Data Availability

The data presented in this study are available on request from the corresponding author. The data are not publicly available due to privacy restrictions.

## Supplementary Materials

The following are available online at https://www.mdpi.com/article/10.3390/toxics9100245/s1, Figure S1. Area Deprivation Index in Flanders (2017) at municipal level, Table S1. Pearson’s correlations between PAH exposure biomarkers and effect biomarkers in Flemish adolescents, Table S2. Detection frequency and limit of quantification (LOQ, µg/L) for OH-PAHs in urine in the fourth Flemish Environment and Health Study (FLEHS-4), Table S3. Significance of associations between study population characteristics and OH-PAHs in univariate analysis, Table S4. Significance of associations between study population characteristics and effect biomarkers in univariate analysis, Table S5. Significance of differences in associations between OH-PAHs and outcomes by sex, Table S6. Linear regression analyses of urinary OH-PAHs concentrations estimated effect for boys in girls of associations that significantly differed by sex, Table S7. Sensitivity analysis, main models of associations between urinary OH-PAHs concentrations and outcomes additionally adjusted for neighborhood socio-economic status, Table S8. Sensitivity analysis, main models of associations between urinary OH-PAHs concentrations and 8-OHdG, leucocyte counts and NLR additionally adjusted for 2-day mean ambient temperature, Table S9. Sensitivity analysis, main models of associations between urinary OH-PAHs and leucocyte counts and NLR additionally adjusted for recent health complaints, Table S10. Associations between HCC, 8-OHdG, leucocyte counts, and NLR and between urinary OH-PAHs concentration and aforementioned outcomes in models adjusted for HCC.
