# Supplementary material for: Urinary Polycyclic Aromatic Hydrocarbon Metabolites Are Associated with Biomarkers of Chronic Endocrine Stress, Oxidative Stress, and Inflammation in Adolescents: FLEHS-4 (2016–2020)
Source: Toxics. 2021 Oct 1;9(10):245. doi: 10.3390/toxics9100245 (PMC8537433; doi:10.3390/toxics9100245)
Supplement: Supplementary file 1 [file toxics-09-00245-s001.zip › toxics-1356629-supplementary.pdf]

Supplementary Materials: Urinary Polycyclic Aromatic Hydrocarbon Metabolites Are Associated with Biomarkers of Chronic Endocrine Stress, Oxidative Stress, and Inflammation in Adolescents: FLEHS-4 (2016–2020)

Veerle J Verheyen, Sylvie Remy, Eva Govarts, Ann Colles, Laura Rodriguez Martin, Gudrun Koppen, Stefan Voorspoels, Liesbeth Bruckers, Esmée M Bijnens, Stijn Vos, Bert Morrens, Dries Coertjens, Annelies De Decker, Carmen Franken, Elly Den Hond, Vera Nelen, Adrian Covaci, Ilse Loots, Stefaan De Henauw, Nicolas Van Larebeke, Caroline Teughels, Tim S Nawrot and Greet Schoeters

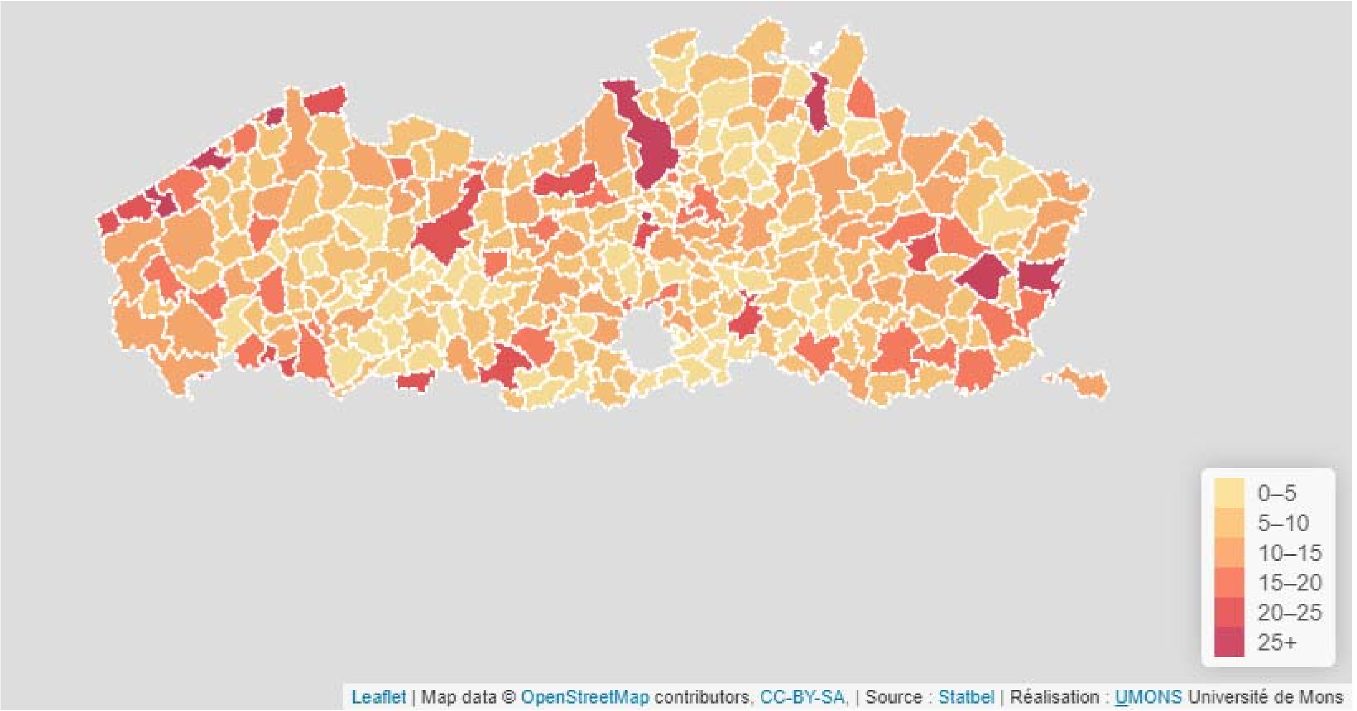

Figure S1. Area Deprivation Index in Flanders at municipal level (2017), according to Lahaye, W., Pannecoucke, I., & Sansen, F. (2019). Kinderarmoede en het lokale niveau—De gemeenten in kaart | Koning Boudewijnstichting. https://www.kbs-frb.be/nl/kinderarmoede_inkaart.

Table S1. Pearson's correlations between PAH exposure biomarkers and effect biomarkers in Flemish adolescents (*n* = 393, except noted differently.

		1-OHPy	2-OHNa	2,3-OHFl	2-OHPH	3-OHPH	1,9-OHPH	ΣOH-PAHs	HCC	Leukocytes	Neutrophils	Lymphocytes	Monocytes	NLR	8-oxodG
**1-OHPy**	Pearson's *r*	**1**													
	n	391													
**2-OHNa**	Pearson's *r*	**0.23**	**1**												
	n	391	392												
**2,3-OHFl**	Pearson's *r*	**0.62**	**0.27**	**1**											
	n	391	392	392											
**2-OHPH**	Pearson's *r*	**0.61**	**0.19**	**0.64**	**1**										
	n	391	392	392	393										
**3-OHPH**	Pearson's *r*	**0.70**	**0.18**	**0.69**	**0.81**	**1**									
	n	391	392	392	393	393									
**1,9-OHPH**	Pearson's *r*	**0.61**	**0.18**	**0.54**	**0.52**	**0.71**	**1**								
	n	391	392	392	393	393	393								
**ΣOH-PAHs**	Pearson's *r*	**0.29**	**0.99**	**0.32**	**0.26**	**0.25**	**0.24**	**1**							
	n	391	391	391	391	391	391	391							
**HCC**	Pearson's *r*	**0.14**	0.02	0.09	0.06	0.06	0.05	0.02	**1**						
	n	391	392	392	393	393	393	391	393						
**Leukocytes**	Pearson's *r*	0.03	0.02	0.07	**0.11**	0.02	-0.02	0.02	0.07	**1**					
	n	391	392	392	393	393	393	391	393	393					
**Neutrophils**	Pearson's *r*	0.08	0.07	**0.11**	**0.12**	0.04	0.02	0.07	**0.12**	**0.90**	**1**				
	n	391	392	392	393	393	393	391	393	393	393				
**Lymphocytes**	Pearson's *r*	-0.06	-0.04	-0.06	-0.02	-0.06	-0.08	-0.05	-0.08	**0.45**	0.09	1			
	n	391	392	392	393	393	393	391	393	393	393	393			
**Monocytes**	Pearson's *r*	0.01	-0.08	-0.01	0.06	0.01	-0.05	-0.07	0.06	**0.57**	**0.41**	**0.30**	1		
	n	391	392	392	393	393	393	391	393	393	393	393	393		
**NLR**	Pearson's *r*	**0.10**	0.09	**0.13**	**0.12**	0.07	0.06	0.09	**0.15**	**0.53**	**0.82**	**-0.49**	**0.19**	1	
	n	391	392	392	393	393	393	391	393	393	393	393	393	393	
**8-oxodG**	Pearson's *r*	**0.10**	0.02	0.03	**0.17**	0.09	-0.09	0.02	-0.05	-0.02	-0.01	-0.04	-0.03	0.02	1
	n	391	392	392	393	393	393	391	393	393	393	393	393	393	393

All biomarkers are ln-transformed. Significant correlations ($p \leq 0.05$) are marked in bold. Urinary biomarkers are normalized for urinary specific gravity. Abbreviations: PAH polycyclic aromatic hydrocarbon, 1-OHPy 1-hydroxypyrene, 2-OHNa 2-hydroxynaphthalene, 2,3-OHFl sum of 2-hydroxyfluorene and 3-hydroxyfluorene, 2-OHPH 2-hydroxyphenanthrene, 3-OHPH 3-hydroxyphenanthrene, 1,9-OHPH sum of 1-hydroxyphenanthrene and 9-hydroxyphenanthrene, ΣOH-PAHs sum of molar concentrations of all measured OH-PAHs, HCC hair cortisol concentration, NLR neutrophil-to-lymphocyte ratio, 8-oxodG 8-oxo-7,8-dihydro-2'-deoxyguanosine.

Table S2. Detection frequency and limit of quantification (LOQ, µg/L) for OH-PAHs in urine in the fourth Flemish Environment and Health Study (FLEHS-4).

Biomarker	LOQ (µg/L)	% > LOQ
**OH-PAHs**		
2-OHNa	0.150	100
2,3-OHFl	0.030	99.5
2-OHPH	0.015	97.6
3-OHPH	0.014	98.8
4-OHPH	0.014	6.9
1,9-OHPH	0.031	98.1
1-OHPy	0.015	97.6

Abbreviations: PAH polycyclic aromatic hydrocarbon, 1-OHPy 1-hydroxypyrene, 2-OHN 2-hydroxynaphthalene, 2,3-OHF sum of 2-hydroxyfluorene and 3-hydroxyfluorene, 2-OHPH 2-hydroxyphenanthrene, 3-OHPH 3-hydroxyphenanthrene, 1,9-OHPH sum of 1-hydroxyphenanthrene and 9-hydroxyphenanthrene, LOQ limit of quantification.

Table S3. Significance of associations between study population characteristics and OH-PAHs in univariate analysis.

	2-OHNa	2,3-OHFl	2-OHPH	3-OHPH	1,9-OHPH	1-OHPy	ΣOH-PAH
**Sex**							
Male	reference	reference	reference	reference	reference	reference	reference
Female	**1.32 (1.11, 1.57)**	1.02 (0.91, 1.14)	0.90 (0.80, 1.00)	0.90 (0.81, 1.00)	1.08 (0.95, 1.22)	1.06 (0.94, 1.19)	**1.27 (1.08, 1.49)**
Age							
< 14.5	reference	reference	reference	reference	reference	reference	reference
14.5–15.5	1.15 (0.94, 1.40)	**1.14 (1.00, 1.24)**	1.13 (0.99, 1.28)	**1.17 (1.04, 1.32)**	**1.28 (1.11, 1.48)**	**1.22 (1.07, 1.40)**	**1.16 (1.11, 1.48)**
> 15.5	1.19 (0.84, 1.69)	**1.35 (1.07, 1.69)**	**1.29 (1.03, 1.61)**	**1.26 (1.02, 1.56)**	1.24 (0.96, 1.59)	1.22 (0.96, 1.53)	1.21 (0.88, 1.68)
Body Mass Index							
Underweight	reference	reference	reference	reference	reference	reference	reference
Normal weight	1.11 (0.81, 1.53)	1.06 (0.86, 1.30)	1.19 (0.97, 1.47)	1.07 (0.88, 1.30)	1.13 (0.90, 1.43)	1.05 (0.86, 1.39)	1.10 (0.82, 1.47)
Overweight, obese	**1.58 (1.10, 2.26)**	**1.30 (1.03, 1.63)**	**1.46 (1.15, 1.84)**	1.06 (0.85, 1.32)	1.13 (0.87, 1.48)	1.09 (0.86, 1.39)	**1.49 (1.07, 2.08)**
Perceived income adequacy							
Difficult	reference	reference	reference	reference	reference	reference	reference
Rather easy	**0.79 (0.63, 0.98)**	**0.85 (0.74, 0.99)**	0.87 (0.76, 1.01)	0.90 (0.78, 1.03)	0.93 (0.79, 1.10)	0.87 (075, 1.01)	**0.79 (0.65, 0.97)**
Easy to very easy	**0.73 (0.59, 0.90)**	**0.79 (0.69, 0.90)**	**0.76 (0.66, 0.87)**	**0.84 (0.74, 0.96)**	0.91 (.78, 1.06)	**0.83 (0.72, 0.96)**	**0.73 (0.60, 0.89)**
Area Deprivation Index							
0–5.3%	reference	reference	reference	reference	reference	reference	reference
5.4–9.3%	0.98 (0.76, 1.26)	1.18 (1.01, 1.38)	1.14 (0.97, 1.34)	1.08 (0.93, 1.26)	0.99 (0.83, 1.19)	1.14 (0.97, 1.35)	0.98 (0.78, 1.23)
9.4–15.5%	1.21 (0.94, 1.54)	**1.23 (1.05, 1.44)**	**1.17 (1.05, 1.38)**	1.12 (0.96, 1.30)	1.03 (0.86, 1.23)	1.08 (0.92, 1.27)	1.21 (0.96, 1.52)
> 15.5%	1.03 (0.80, 1.31)	1.09 (0.93, 1.28)	**1.22 (1.04, 1.43)**	1.11 (0.95, 1.29)	0.95 (0.79, 1.14)	1.04 (0.89, 1.23)	1.03(0.82, 1.29)
Smoking							
No	reference	reference	reference	reference	reference	reference	reference
Yes	**1.88 (1.23, 2.88)**	2.49 (1.92, 3.23)	2.27 (0.97, 1.67)	1.27 (0.98, 1.64)	1.28 (0.94, 1.72)	1.24 (0.93, 1.66)	**1.81 (1.21, 2.70)**
Residential exposure to ETS							
No	reference	reference	reference	reference	reference	reference	reference
Yes	1.28 (0.96, 1.70)	**1.36 (1.14, 1.64)**	**1.34 (1.12, 1.62)**	**1.22 (1.03, 1.45)**	1.07 (0.87, 1.31)	**1.09 (1.06, 1.56)**	1.25 (0.96, 1.63)
Season							
Winter	reference	reference	reference	reference	reference	reference	reference
Spring	0.84 (0.69, 1.03)	1.14 (1.02, 1.29)	**1.30 (1.14, 1.47)**	**1.22 (1.08, 1.37)**	**1.16 (1.01, 1.34)**	**1.14 (1.00, 1.30)**	0.86 (0.72, 1.04)
Summer	-	-	-	-	-	-	-
Fall	0.96 (0.76, 1.22)	1.08 (0.92, 1.26)	1.13 (0.97, 1.31)	0.98 (0.85, 1.13)	0.80 (0.67, 0.95)	0.96 (0.82, 1.12)	0.96 (0.77, 1.20)
2-day mean temperature (°C)							
< 6	reference	reference	reference	reference	reference	reference	reference
6–12	0.83 (1.68, 1.02)	1.02 (0.90, 1.16)	1.10 (0.98, 1.26)	1.03 (0.91, 1.17)	0.98 (0.84, 1.14)	0.95 (0.86, 1.09)	0.85 (0.70, 1.03)
> 12	0.85 (0.68, 1.05)	**1.22 (1.06, 1.40)**	**1.36 (1.19, 1.56)**	**1.29 (1.14, 1.47)**	1.15 (0.98, 1.35)	1.14 (0.98, 1.32)	0.86 (0.71, 1.06)
Recent health complaints							
No	reference	reference	reference	reference	reference	reference	reference
Yes	1.09 (0.90, 1.32)	0.97 (0.86, 1.10)	0.98 (0.87, 1.11)	0.95 (0.85, 1.07)	0.92 (0.80, 1.06)	0.97 (0.86, 1.10)	1.08 (0.90, 1.29)

All biomarkers are ln-transformed. Significant correlations ($p \leq 0.05$) are marked in bold. Abbreviations: OH-PAHs hydroxylated polycyclic aromatic hydrocarbon, 2-OHNa 2-hydroxy-naphthalene, 2,3-OHFl sum of 2-hydroxy-fluorene and 3-hydroxy-fluorene, 2-OHPH 2-hydroxy-phenanthrene, 3-OHPH 3-hydroxy-phenanthrene, 1,9-OHPH sum of 1-hydroxy-phenanthrene and 9-hydroxy-phenanthrene, 1-OHPy 1-hydroxy-pyrene, ΣOH-PAHs sum of molar concentrations of all measured OH-PAHs, ETS environmental tobacco smoke.

Table S4. Significance of associations between study population characteristics and effect biomarkers in univariate analysis.

	**HCC**	**Leucocytes**	**Neutrophils**	**Lymphocytes**	**Monocytes**	**NLR**	**8-oxodG**
**Sex**							
Male	reference	reference	reference	reference	reference	reference	reference
Female	1.14 (0.98, 1.33)	**1.08 (1.03, 1.13)**	**1.21 (1.13, 1.31)**	0.96 (0.92, 1.01)	0.97 (0.92, 1.03)	**1.26 (1.16, 1.387)**	1.01 (0.93, 1.10)
Age							
< 14.5	reference	reference	reference	reference	reference	reference	reference
14.5–15.5	1.13 (0.95, 1.35)	1.04 (0.99, 1.10)	1.09 (1.00, 1.09)	1.00 (0.95, 1.06)	1.03 (0.96, 1.10)	1.09 (0.98, 1.20)	1.02 (0.92, 1.13)
> 15.5	**1.39 (1.03, 1.89)**	1.10 (1.00, 1.21)	**1.17 (1.01, 1.36)**	1.01 (0.92, 1.12)	0.98 (0.87, 1.10)	1.15 (0.97, 1.37)	1.14 (0.96, 1.35)
Body Mass Index							
Underweight	reference	reference	reference	reference	reference	reference	reference
Normal weight	1.08 (0.82, 1.43)	1.00 (0.91, 1.09)	0.97 (0.85, 1.12)	1.05 (0.96, 1.15)	0.98 (0.88, 1.09)	0.92 (0.79, 1.08)	1.00 (0.85, 1.17)
Overweight, obese	1.21 (0.88, 1.66)	1.06 (0.96, 1.18)	1.10 (0.94, 1.29)	1.06 (0.95, 1.17)	1.00 (0.87, 1.11)	1.05 (0.87, 1.25)	0.98 (0.82, 1.17)
Perceived Income Adequacy							
Difficult	reference	reference	reference	reference	reference	reference	reference
Rather easy	0.88 (0.72, 1.07)	1.02 (0.96, 1.09)	1.00 (0.90, 1.10)	1.05 (0.98, 1.123	1.03 (0.96, 1.11)	0.95 (0.85, 1.06)	0.94 (0.84, 1.05)
Easy to very easy	0.82 (0.68, 1.00)	1.01 (0.95, 1.07)	0.98 (0.90, 1.08)	1.02 (0.96, 1.09)	1.00 (0.93, 1.07)	0.96 (0.86, 1.07)	0.95 (0.85, 1.06)
Area Deprivation Index							
0–5.3%	reference	reference	reference	reference	reference	reference	reference
5.4–9.3%	0.94 (0.76, 1.17)	1.01 (0.94, 1.058)	1.00 (0.90, 1.12)	1.00 (0.93, 1.07)	0.9 (0.91, 1.07)	1.00 (0.89, 1.14)	1.03 (0.91, 1.17)
9.4–15.5%	1.03 (0.83, 1.28)	0.97 (0.91, 1.04)	0.98 (0.88, 1.09)	0.96 (0.90, 1.03)	0.97 (0.89, 1.05)	1.01 (0.90, 1.15)	1.02 (0.90, 1.15)
> 15.5%	1.02 (0.82, 1.27)	1.04 (0.97, 1.11)	1.05 (0.94, 1.17)	1.00 (0.93, 1.07)	1.03 (0.95, 1.12)	1.06 (0.93, 1.20)	1.01 (0.89, 1.14)
Smoking							
No	reference	reference	reference	reference	reference	reference	reference
Yes	1.11 (0.77, 1.59)	1.03 (0.92, 1.16)	1.05 (0.87, 1.26)	1.01 (0.90, 1.14)	1.00 (0.87, 1.15)	1.03 (0.84, 1.27)	1.15 (0.93, 1.41)
Residential exposure to ETS							
No	reference	reference	reference	reference	reference	reference	reference
Yes	0.83 (0.64, 1.07)	**1.09 (1.01, 1.19)**	1.10 (0.97, 1.25)	**1.10 (1.01, 1.19)**	1.03 (0.94, 1.14)	1.00 (0.86, 1.15)	**1.21 (1.05, 1.39)**
Season							
Winter	reference	reference	reference	reference	reference	reference	reference
Spring	1.10 (0.92, 1.31)	1.02 (0.96, 1.08)	1.00 (0.92, 1.09)	1.05 (0.99, 1.11)	1.01 (0.94, 1.08)	0.95 (0.86, 1.05)	0.99 (0.90, 1.410)
Summer	-	-	-	-	-	-	-
Fall	1.21 (0.99, 1.50)	1.02 (0.96, 1.09)	1.03 (0.93, 1.15)	0.98 (0.91, 1.04)	1.01 (0.93, 1.09)	1.06 (0.94, 1.19)	1.08 (0.96, 1.22)
2-day mean temp							
<6	-	reference	reference	reference	reference	reference	reference
6–12	-	1.02 (0.96, 1.08)	1.03 (0.94, 1.12)	0.99 (0.93, 1.05)	1.00 (0.93, 1.07)	1.04 (0.94, 1.15)	1.00 (0.91, 1.11)
> 12	-	0.98 (0.92, 1.04)	0.92 (0.81, 1.01)	1.06 (0.99, 1.12)	1.00 (0.93, 1.08)	**0.87 (0.78, 0.97)**	0.96 (0.86, 1.07)
Recent health complaints							
No	-	reference	reference	reference	reference	reference	reference
Yes	-	**1.09 (1.04, 1.15)**	**1.14 (1.05, 1.23)**	1.01 (0.93, 1.07)	**1.09 (1.02, 1.16)**	**1.12 (1.02, 1.24)**	1.02 (0.93, 1.13)

All biomarkers are ln-transformed. Significant correlations ($p \leq 0.05$) are marked in bold. 8-OHdG is adjusted for urinary specific gravity. ETS environmental tobacco smoke, HCC hair cortisol concentration, NLR neutrophil to lymphocyte ratio, 8-oxodG 8-oxo-7,8-dihydro-2'-deoxyguanosine.

Table S5. Significance of differences in associations between OH-PAHs and outcomes by sex.

	***p*-value of interaction by sex**						
**OH-PAHs**	**HCC**	**Leucocytes**	**Neutrophils**	**Lymphocytes**	**Monocytes**	**NLR**	**8-oxodG**
2-OHNa	**0.067**	0.240	0.271	0.384	0.570	0.649	0.449
2,3-OHFl	**0.154**	0.239	0.979	**0.012**	0.305	**0.147**	0.991
2-OHPH	**0.079**	0.706	0.920	0.477	0.310	0.739	0.697
3-OHPH	0.235	0.700	0.942	0.456	0.630	0.616	0.940
1,9-OHPH	0.485	0.325	0.204	0.602	0.202	0.205	0.300
1-OHPy	0.642	0.264	0.544	0.407	0.447	0.963	0.799
ΣOH-PAH	0.580	0.849	0.625	0.470	0.275	0.398	0.332

Significance of the interaction term of OH-PAH and sex in models, adjusted for sex, age, BMI, household socio-economic status, season of sampling, smoking and residential exposure to environmental tobacco smoke is presented. Significant interactions (*p*-interaction ≤ 0.20) are marked in bold. Abbreviations: OH-PAHs hydroxylated polycyclic aromatic hydrocarbon, 1-OHPy 1-hydroxypyrene, 2-OHNa 2-hydroxynaphthalene, 2,3-OHFl sum of 2-hydroxyfluorene and 3-hydroxyfluorene, 2-OHPH 2-hydroxyphenanthrene, 3-OHPH 3-hydroxyphenanthrene, 1,9-OHPH sum of 1-hydroxyphenanthrene and 9-hydroxyphenanthrene, ΣOH-PAHs sum of molar concentrations of all measured OH-PAHs, HCC hair cortisol concentration, NLR neutrophil-to-lymphocyte ratio, 8-oxodG 8-oxo-7,8-dihydro-2'-deoxyguanosine.

Table S6. Linear regression analyses of urinary OH-PAHs concentrations, estimated effect for boys in girls of associations that significantly differed by sex.

	**Boys**	**Girls**
**OH-PAHs**	**β (95% CI)**	**β (95% CI)**
	**HCC (pg/mg)**	
2-OHNa	1.05 (0.96, 1.15)	0.93 (0.86, 1.02)
2,3-OHFl	1.13 (0.98, 1.30)	0.99 (0.87, 1.14)
2-OHPH	1.11 (0.96, 1.28)	0.95 (0.84, 1.08)
	**Lymphocytes (cells/μL)**	
2,3-OHFl	1.02 (0.97, 1.07)	**0.94 (0.90, 0.98)**
	**neutrophil-to-lymphocyte ratio (NLR)**	
2,3-OHFl	1.02 (0.94, 1.10)	**1.10 (1.02, 1.18)**

Effect estimates β are presented with their 95% confidence interval (95% CI) as the factor change in hair cortisol concentration (HCC), lymphocyte count and neutrophil-to-lymphocyte ratio (NLR) for a doubling in OH-PAH concentration Models adjusted for sex, age, BMI, household socio-economic status, season of sampling, smoking and residential exposure to environmental tobacco smoke and including the interaction term of each OH-PAH with sex. Significant associations are marked in bold. Abbreviations: OH-PAHs hydroxylated polycyclic aromatic hydrocarbon, 1-OHPy 1-hydroxypyrene, 2-OHNa 2-hydroxynaphthalene, 2,3-OHFl sum of 2-hydroxyfluorene and 3-hydroxyfluorene, 2-OHPH 2-hydroxyphenanthrene, 3-OHPH 3-hydroxyphenanthrene, 1,9-OHPH sum of 1-hydroxyphenanthrene and 9-hydroxyphenanthrene.

Table S7. Sensitivity analysis, main models of associations between urinary OH-PAHs concentrations and outcomes additionally adjusted for neighborhood socio-economic status.

	β (95% CI)						
**OH-PAHs**	**HCC**	**Leucocytes**	**Neutrophils**	**Lymphocytes**	**Monocytes**	**NLR**	**8-oxodG**
2-OHNa	0.98 (0.92, 1.05)	1.00 (0.98, 1.02)	1.00 (0.97, 1.04)	0.99 (0.97, 1.01)	0.98 (0.96, 1.00)	1.01 (0.98, 1.05)	1.01 (0.98, 1.05)
2,3-OHFl	1.06 (0.95, 1.18)	1.01 (0.98, 1.05)	1.04 (0.99, 1.09)	0.97 (0.94, 1.01)	1.00 (0.96, 1.04)	**1.07 (1.01, 1.13)**	1.04 (0.98, 1.10)
2-OHPH	1.03 (0.93, 1.14)	**1.03 (1.00, 1.06)**	**1.06 (1.01, 1.11)**	0.98 (0.95, 1.01)	1.02 (0.98, 1.06)	**1.08 (1.02, 1.14)**	**1.08 (1.02, 1.14)**
3-OHPH	1.06 (0.95, 1.17)	1.01 (0.97, 1.04)	1.02 (0.97, 1.08)	0.97 (0.94, 1.00)	1.00 (0.96, 1.05)	1.06 (1.00, 1.12)	**1.06 (1.00, 1.12)**
1,9-OHPH	1.02 (0.94, 1.12)	0.99 (0.96, 1.02)	1.00 (0.96, 1.04)	**0.97 (0.94, 1.00)**	0.99 (0.95, 1.02)	1.03 (0.98, 1.03)	1.01 (0.96, 1.06)
1-OHPy	**1.13 (1.03, 1.25)**	1.00 (0.97, 1.03)	1.02 (0.97, 1.07)	0.98 (0.95, 1.01)	1.00 (0.97, 1.04)	1.05 (0.99, 1.10)	**1.07 (1.02, 1.13)**
ΣOH-PAHs	0.98 (0.91, 1.05)	1.00 (0.98, 1.02)	1.01 (0.98, 1.04)	0.99 (0.97, 1.01)	0.98 (0.96, 1.01)	1.02 (0.98, 1.06)	1.02 (0.98, 1.06)

Effect estimates β are presented with their 95% confidence interval (95% CI) as the factor change in HCC, leucocyte count or NLR for a doubling in OH-PAH concentration. Models are adjusted for sex, age, BMI, household socio-economic status, neighborhood socio-economic status, season, smoking and residential exposure to environmental tobacco smoke. Significant associations (*p*-value ≤ 0.05) are marked in bold. Abbreviations: OH-PAHs hydroxylated polycyclic aromatic hydrocarbon, 1-OHPy 1-hydroxypyrene, 2-OHNa 2-hydroxynaphthalene, 2,3-OHFl sum of 2-hydroxyfluorene and 3-hydroxyfluorene, 2-OHPH 2-hydroxyphenanthrene, 3-OHPH 3-hydroxyphenanthrene, 1,9-OHPH sum of 1-hydroxyphenanthrene and 9-hydroxyphenanthrene, ΣOH-PAHs sum of molar concentrations of all measured OH-PAHs, HCC hair cortisol concentration, 8-oxodG 8-oxo-7,8-dihydro-2'-deoxyguanosine, NLR neutrophil-to-lymphocyte ratio.

Table S8. Sensitivity analysis, main models of associations between urinary OH-PAHs concentrations and 8-OHdG, leucocyte counts and NLR additionally adjusted for 2-day mean ambient temperature.

	β (95% CI)					
**OH-PAHs**	**Leucocytes**	**Neutrophils**	**Lymphocytes**	**Monocytes**	**NLR**	**8-oxodG**
2-OHNa	1.00 (0.98, 1.02)	1.01 (0.97, 1.04)	0.99 (0.97, 1.01)	0.98 (0.96, 1.00)	1.01 (0.98, 1.05)	1.01 (0.98, 1.05)
2,3-OHFl	1.01 (0.98, 1.05)	1.04 (0.99, 1.09)	0.97 (0.94, 1.00)	1.00 (0.96, 1.04)	**1.07 (1.01, 1.14)**	1.05 (0.99, 1.11)
2-OHPH	**1.03 (1.00, 1.07)**	**1.07 (1.01, 1.12)**	0.98 (0.95, 1.01)	1.02 (0.98, 1.06)	**1.09 (1.03, 1.15)**	**1.08 (1.02, 1.14)**
3-OHPH	1.01 (0.97, 1.04)	1.03 (0.98, 1.08)	**0.97 (0.93, 1.00)**	1.00 (0.96, 1.04)	**1.06 (1.00, 1.13)**	**1.06 (1.00, 1.12)**
1,9-OHPH	0.99 (0.96, 1.01)	0.99 (0.95, 1.04)	0.97 (0.94, 1.00)	0.98 (0.95, 1.02)	1.02 (0.97, 1.07)	1.01 (0.96, 1.06)
1-OHPy	1.00 (0.97, 1.03)	1.02 (0.97, 1.07)	0.97 (0.94, 1.00)	1.00 (0.97, 1.04)	1.05 (0.99, 1.11)	**1.07 (1.02, 1.13)**
ΣOH-PAHs	1.00 (0.98, 1.02)	1.01 (0.98, 1.04)	0.99 (0.97, 1.01)	0.98 (0.96, 1.01)	1.02 (0.98, 1.06)	1.02 (0.98, 1.06)

Effect estimates β are presented with their 95% confidence interval (95% CI) as the factor change in leucocyte count or NLR for a doubling in OH-PAH concentration. Models are adjusted for sex, age, BMI, household socio-economic status, season of sampling, smoking and residential exposure to environmental tobacco smoke, 2-day mean ambient temperature. Models for 8-OHdG are additionally adjusted for urinary density. Significant associations (*p*-value ≤ 0.05) are marked in bold. Abbreviations: OH-PAHs hydroxylated polycyclic aromatic hydrocarbon, 1-OHPy 1-hydroxypyrene, 2-OHNa 2-hydroxynaphthalene, 2,3-OHFl sum of 2-hydroxyfluorene and 3-hydroxyfluorene, 2-OHPH 2-hydroxyphenanthrene, 3-OHPH 3-hydroxyphenanthrene, 1,9-OHPH sum of 1-hydroxyphenanthrene and 9-hydroxyphenanthrene, ΣOH-PAHs sum of molar concentrations of all measured OH-PAHs, 8-oxodG 8-oxo-7,8-dihydro-2'-deoxyguanosine, NLR neutrophil-to-lymphocyte ratio.

Table S9. Sensitivity analysis, main models of associations between urinary OH-PAHs and leucocyte counts and NLR additionally adjusted for recent health complaints.

	β (95% CI)				
**OH-PAHs**	**Leucocytes**	**Neutrophils**	**Lymphocytes**	**Monocytes**	**NLR**
2-OHNa	1.00 (0.98, 1.02)	1.00 (0.97, 1.04)	0.99 (0.97, 1.01)	0.98 (0.96, 1.00)	1.01 (0.98, 1.05)
2,3-OHFl	1.01 (0.98, 1.04)	1.03 (0.98, 1.09)	0.97 (0.94, 1.00)	1.00 (0.96, 1.04)	**1.07 (1.01, 1.13)**
2-OHPH	**1.03 (1.00, 1.06)**	**1.06 (1.01, 1.11)**	0.98 (0.95, 1.01)	1.02 (0.98, 1.06)	**1.08 (1.02, 1.14)**
3-OHPH	1.00 (0.97, 1.04)	1.02 (0.97, 1.07)	**0.97 (0.94, 1.00)**	1.00 (0.96, 1.04)	**1.06 (1.00, 1.12)**
1,9-OHPH	0.99 (0.96, 1.02)	1.00 (0.95, 1.04)	**0.97 (0.94, 1.00)**	0.99 (0.95, 1.02)	1.03 (0.98, 1.08)
1-OHPy	1.00 (0.97, 1.03)	1.02 (0.97, 1.07)	0.97 (0.94, 1.01)	1.00 (0.97, 1.04)	1.04 (0.99, 1.10)
ΣOH-PAHs	1.00 (0.98, 1.02)	1.01 (0.97, 1.04)	0.99 (0.97, 1.01)	0.98 (0.96, 1.01)	1.02 (0.98, 1.06)

Effect estimates β are presented with their 95% confidence interval (95% CI) as the factor change in leucocyte count or NLR for a doubling in OH-PAH concentration. Models are adjusted for sex, age, BMI, household socio-economic status, season of sampling, smoking and residential exposure to environmental tobacco smoke, recent health complaints. Significant associations (p-value ≤ 0.05) are marked in bold. Abbreviations: OH-PAHs hydroxylated polycyclic aromatic hydrocarbon, 1-OHPy 1-hydroxypyrene, 2-OHNa 2-hydroxynaphthalene, 2,3-OHFl sum of 2-hydroxyfluorene and 3-hydroxyfluorene, 2-OHPH 2-hydroxyphenanthrene, 3-OHPH 3-hydroxyphenanthrene, 1,9-OHPH sum of 1-hydroxyphenanthrene and 9-hydroxyphenanthrene, ΣOH-PAHs sum of molar concentrations of all measured OH-PAHs, NLR neutrophil-to-lymphocyte ratio.

Table S10. Associations between HCC, 8-OHdG, leucocyte counts and NLR and between urinary OH-PAHs concentration and aforementioned outcomes in models adjusted for HCC.

	β (95% CI)					
	**Leucocytes**	**Neutrophils**	**Lymphocytes**	**Monocytes**	**NLR**	**8-oxodG**
HCC	1.01 (0.99, 1.03)	1.03 (0.99,1.07)	0.98 (0.96, 1.01)	1.02 (0.99, 1.05)	**1.05 (1.01, 1.09)**	0.98 (0.94, 1.02)
2-OHNa	1.00 (0.98, 1.02)	1.01 (0.97, 1.04)	0.99 (0.97, 1.01)	0.98 (0.96, 1.01)	1.01 (0.98, 1.05)	1.01 (0.98, 1.05)
HCC	1.01 (0.99, 1.03)	1.03 (0.99, 1.07)	0.98 (0.96 1.01)	1.02 (0.96, 1.04)	**1.05 (1.01, 1.09)**	0.98 (0.94, 1.02)
2,3-OHFl	1.01 (0.98, 1.04)	1.03 (0.98, 1.08)	0.97 (0.94, 1.00)	1.00 (0.96, 1.04)	**1.06 (1.00, 1.12)**	1.05 (0.99, 1.11)
HCC	1.01 (0.99, 1.03)	1.03 (0.99, 1.07)	0.99 (0.96 1.01)	1.02 (0.96, 1.04)	**1.04 (1.00, 1.09)**	0.98 (0.94, 1.01)
2-OHPH	**1.03 (1.00, 1.06)**	**1.06 (1.01, 1.11)**	0.98 (0.95, 1.01)	1.02 (0.98, 1.06)	**1.08 (1.02, 1.14)**	**1.08 (1.02, 1.14)**
HCC	1.01 (0.99, 1.03)	1.03 (0.99, 1.07)	0.99 (0.96 1.01)	1.02 (0.96, 1.04)	**1.05 (1.01, 1.09)**	0.98 (0.94, 1.01)
3-OHPH	1.00 (0.97, 1.04)	1.02 (0.97, 1.07)	**0.97 (0.94, 1.00)**	1.00 (0.96, 1.04)	1.05 (0.99, 1.11)	**1.06 (1.00, 1.12)**
HCC	1.01 (0.99, 1.03)	1.03 (0.99, 1.07)	0.99 (0.96 1.01)	1.02 (0.96, 1.04)	**1.04 (1.00, 1.09)**	0.98 (0.94, 1.01)
1,9-OHPH	0.99 (0.96, 1.01)	0.99 (0.95, 1.04)	**0.97 (0.94, 1.00)**	0.98 (0.95, 1.02)	1.02 (0.97, 1.07)	1.01 (0.96, 1.06)
HCC	1.01 (0.99, 1.03)	1.03 (0.99, 1.07)	0.99 (0.96 1.01)	1.02 (0.96, 1.04)	**1.05 (1.01, 1.09)**	0.98 (0.94, 1.02)
1-OHPy	1.00 (0.97, 1.03)	1.01 (0.96, 1.01)	0.98 (0.95, 1.01)	1.00 (0.96, 1.04)	1.04 (0.98, 1.09)	**1.08 (1.02, 1.13)**
HCC	1.01 (0.99, 1.03)	1.03 (0.99, 1.07)	0.99 (0.96, 1.01)	1.02 (0.96, 1.04)	**1.04 (1.00, 1.09)**	0.97 (0.93, 1.01)
ΣOH-PAHs	1.00 (0.98, 1.02)	1.01 (0.98, 1.05)	0.99 (0.97, 1.01)	0.98 (0.96, 1.01)	1.02 (0.98, 1.06)	1.02 (0.98, 1.06)
HCC	1.01 (0.99, 1.03)	1.03 (0.99, 1.07)	0.98 (0.96 1.01)	1.02 (0.96, 1.04)	**1.05 (1.01, 1.09)**	0.98 (0.94, 1.02)

Effect estimates β are presented with their 95% confidence interval (95% CI) as the factor change in leucocyte count or NLR for a doubling in OH-PAH concentration. Models are adjusted for sex, age, BMI, household socio-economic status, season of sampling, smoking and residential exposure to environmental tobacco smoke. Significant associations (p-value ≤ 0.05) are marked in bold. Abbreviations: PAH polycyclic aromatic hydrocarbon, 1-OHPy 1-hydroxypyrene, 2-OHNa 2-hydroxynaphthalene, 2,3-OHFl sum of 2-hydroxyfluorene and 3-hydroxyfluorene, 2-OHPH 2-hydroxyphenanthrene, 3-OHPH 3-hydroxyphenanthrene, 1,9-OHPH sum of 1-hydroxyphenanthrene and 9-hydroxyphenanthrene, ΣOH-PAHs sum of molar concentrations of all measured OH-PAHs, 8-oxodG 8-oxo-7,8-dihydro-2'-deoxyguanosine, NLR neutrophil-to-lymphocyte ratio, HCC hair cortisol concentration.
